# Nicotine restores olfactory function by activation of prok2R/Akt/FoxO3a axis in Parkinson’s disease

**DOI:** 10.1186/s12967-024-05171-1

**Published:** 2024-04-12

**Authors:** Qinglong Guo, Yi Wang, Liangchen Yu, Liao Guan, Xuefei Ji, Xiaohui Li, Gang Pang, Zhenhua Ren, Lei Ye, Hongwei Cheng

**Affiliations:** 1 Department of Neurosurgery, the First Affiliated Hospital of Anhui Medical University, Jixi Road 218, Hefei, 230022 Anhui China; 2 Department of Anatomy, Anhui Medical University, Meishan Road 81, Hefei, 230032 China; 3 Anhui Provincial Key Laboratory for Brain Bank Construction and Resource Utilization, Meishan Road 81, Hefei, 230032 China

**Keywords:** Parkinson’s disease, Nicotine, Olfactory bulb, Prok2R/Akt/FoxO3a signaling pathway, Apoptosis

## Abstract

**Background:**

Olfactory dysfunction occurs frequently in Parkinson’s disease (PD). In this study, we aimed to explore the potential biomarkers and underlying molecular pathways of nicotine for the treatment of olfactory dysfunction in 1-methyl-4-phenyl-1, 2, 3, 6-tetrahydropyridine (MPTP)-induced PD mice.

**Methods:**

MPTP was introduced into C57BL/6 male mice to generate a PD model. Regarding in vivo experiments, we performed behavioral tests to estimate the protective effects of nicotine in MPTP-induced PD mice. RNA sequencing and traditional molecular methods were used to identify molecules, pathways, and biological processes in the olfactory bulb of PD mouse models. Then, in vitro experiments were conducted to evaluate whether nicotine can activate the prok2R/Akt/FoxO3a signaling pathway in both HEK293T cell lines and primary olfactory neurons treated with 1-methyl-4-phenylpyridinium (MPP^+^). Next, prok2R overexpression (prok2R^+^) and knockdown (prok2R^−^) were introduced with lentivirus, and the Akt/FoxO3a signaling pathway was further explored. Finally, the damaging effects of MPP^+^ were evaluated in prok2R overexpression (prok2R^+^) HEK293T cell lines.

**Results:**

Nicotine intervention significantly alleviated olfactory and motor dysfunctions in mice with PD. The prok2R/Akt/FoxO3a signaling pathway was activated after nicotine treatment. Consequently, apoptosis of olfactory sensory neurons was significantly reduced. Furthermore, prok2R^+^ and prok2R^−^ HEK293T cell lines exhibited upregulation and downregulation of the Akt/FoxO3a signaling pathway, respectively. Additionally, prok2R^+^ HEK293T cells were resistant to MPP^+^-induced apoptosis.

**Conclusions:**

This study showed the effectiveness and underlying mechanisms of nicotine in improving hyposmia in PD mice. These improvements were correlated with reduced apoptosis of olfactory sensory neurons via activated prok2R/Akt/FoxO3a axis. These results explained the potential protective functions of nicotine in PD patients.

**Supplementary Information:**

The online version contains supplementary material available at 10.1186/s12967-024-05171-1.

## Introduction

Parkinson's disease (PD) is one of the most prevalent chronic neurodegenerative disorders involved in central nervous system that causes symptoms including a range of motor and non-motor manifestations. Motor-related symptoms mainly comprise tremors, rigidity, bradykinesia, and postural instability [1]. However, non-motor symptoms may sometimes be ignored but usually precede the onset of motor manifestations, including constipation, anosmia, and rapid eye movement behavior disorder. The pathogenesis of PD is still not fully elucidated and is believed to involve both genetic and environmental factors. Approximately 90% of patients with PD manifest hyposmia, and the olfactory differential test is often clinically applied to predict the onset of PD at an earlier stage (usually Braak stages 1 and 2) [2]. Previous reports have revealed that the olfactory bulb (OB) participates in the early transmission of α-synuclein, which may be associated with olfactory dysfunction [3, 4]. In clinical studies, a meta-analysis showed that the size of the OB was smaller in patients with PD than in healthy individuals [5]. Diffusion tensor imaging analysis also confirmed that the fractional anisotropy value decreased significantly in patients with PD compared with healthy individuals [6]. Therefore, OB may be a key element that plays a pivotal role in the pathogenesis of PD, especially in the early stage.

The OB receives sensory information, such as odors, from olfactory receptor neurons, which are specialized cells located in the nasal cavity and synapse to mitral/tufted cells in the glomerular layer[7]. Epidemiological studies have reported that cigarette smokers had a 30–40% lower risk of PD than never smokers [8]. Therefore, cigarette smoking was considered a protective element against PD [9]. Several studies have reported that nicotine could prevent the degeneration of dopaminergic (DA) neurons in 1-methyl-4-phenyl-1, 2, 3, 6-tetrahydropyridine (MPTP) PD model [10, 11]. However, the detailed mechanisms of the protective effect that nicotine exerts in the pathogenesis of PD through the OB remains unclear.

Recently, a clinical study found that prokineticin-2 (prok2) expression was significantly increased in olfactory neurons among patients with PD and was significantly correlated with the level of oligomeric α-synuclein [12]. Prok2R, a G protein-coupled receptor that binds to the ligands prok2 and prok1 [13], is involved in various physiological processes, including olfactory function [14], circadian rhythm regulation [15], pleasant touch sensation [16], and gastrointestinal function [17]. The prok2/prok2R pathway was found to be associated with OB neurogenesis [18], and knockout of the prok2R gene in mice manifested deficiency in glomerular layer cells in the OB [15]. Gordon et al. reported that prok2 played a protective role by activation of p-Akt and p-Erk in DA neuronal degeneration in the early stage of MPTP-induced PD mice [19]. Nevertheless, the prok2/prok2R pathway in the OB and related olfactory function of nicotine in PD have not been fully elucidated in previous studies.

We have observed the apoptosis of the glomeruli layer cells in MPTP PD cynomolgus monkey model in our previous research [20]. In this study, the preliminary tests revealed that prok2R was upregulated in the OB tissues of nicotine-treated MPTP PD mice as compared to vehicle-treated MPTP PD mice. Based on these findings, we hypothesized that nicotine may exert a neuroprotective role in PD by activating the prok2R/Akt/FoxO3a axis in the olfactory neurons of the OB. Thus, we aimed to investigate the potential biomarkers and underlying molecular pathways of nicotine in the treatment of olfactory dysfunction in MPTP-induced PD mice.

## Materials and methods

### Chemicals and reagents

Nicotine (GC14043) and 1-methyl-4-phenylpyridinium (MPP^+^) iodide (GC18188) were purchased from GlpBio (CA, USA). MPTP (CAS:23,007-85-4) was purchased from Aladdin Biochemical Technology Co., Ltd. Ipatasertib (HY-15186; Shanghai, China) was delivered by MedCheExpress. Dulbecco’s modified Eagle’s medium and phosphate-buffered saline (pH 7.4) were obtained from Basal Media (Shanghai, China). The Wisent (Wisent, Canada) company provided Fetal bovine serum. Penicillin–streptomycin solution (100 ×) and trypsin solution were purchased from Biosharp (Hefei, China). Immunostaining fix solution (P0098), immunostaining permeabilization solution containing Triton X-100 (P0096), protease inhibitor cocktail for general use (100× , P1005), phenylmethanesulfonyl fluoride (ST505), phosphatase inhibitor cocktail for general use (50× , P1045), bicinchoninic acid protein assay kit (P0012), radioimmunoprecipitation assay lysis buffer (RIPA, P0013B), sodium dodecyl sulfate polyacrylamide gel electrophoresis loading buffer (P0015), Cy3-labeled goat anti-rabbit IgG (H + L) (A0516), Cy3-labeled goat anti-mouse IgG (H + L) (A0521), fluorescein isothiocyanate (FITC)-labeled goat anti-rabbit IgG (H + L) (A0562), and antifade mounting medium with 4′,6-diamidino-2-phenylindole (DAPI) (P0131) were purchased from Beyotime (Shanghai, China). The Spark Jade (Qingdao, China) provided SparkZol Reagent (AC0101), SPARKscript II RT Plus Kit (AG0304), and 2XSYBR Green qPCR Mix (AH0104). Antibodies against glyceraldehyde 3-phosphate dehydrogenase (GAPDH; P04406) and horseradish peroxidase (HRP)-conjugated secondary antibodies (D110058, D110087) were purchased from Sangon Biotech (Shanghai, China). Anti-tyrosine hydroxylase (TH) (ab137869) and anti-prokineticin 2 (ab76747) antibodies were purchased from Abcam (MA, USA). Cleaved-caspase 3 (Cl-casp3) (Asp175) (5A1E) rabbit mAb (9664) was purchased from Cell Signaling Technology (MA, USA). The PRKAR2A pAb (10,142–2-AP), FoxO3a mAb (66,428–1-Ig), Bax pAb (50,599–2-Ig), and Bcl-2 pAb (26,593–1-AP) were obtained from Proteintech (Wuhan, China). Phospho-Akt-S473 rabbit mAb (AP1208) and pan-Akt rabbit pAb (A18120) were obtained from ABclonal (Wuhan, China). Phospho-FoxO3a (Ser253) rabbit mAb (R24347) was purchased from Zenbio (NC, USA). OMP (sc-365818) was purchased from Santa Cruz Biotechnology (TX, USA).

### In vivo experiments

Six-week-old male C57BL/6 mice were provided by the Animal Experiment Center of Anhui Medical University (Hefei, China). These mice were housed with free access to food and water under a 12/12 h light/dark cycle at the room temperature of 24 °C. A randomization number table was used to allocate the mice to different treatment groups. The in vivo experiments were performed according to the regulations suggested in the Animal Research: Reporting of In Vivo Experiments (ARRIVE) guidelines. Based on the previous experimental results for this platform, the effect size *f* was 0.85. The two sizes were set at α = 0.05, and the unilateral test efficacy β was 0.9. The number of mice in each group was defined using G*power [21]. The number was calculated to be 7.6 in each group, providing 90% power with a type I error α of 0.05. Therefore, 8 mice were randomly enrolled in each group: normal control group injected with vehicle (Vehicle), small dosage preventative nicotine (0.1 mg/kg daily) group (MPTP + Nic_S), large dosage preventative nicotine (1 mg/kg daily) group (MPTP + Nic_L), large dosage therapeutic (1 mg/kg daily) group (MPTP + Nic_T), and MPTP PD model group (MPTP + Vehicle). MPTP (30 mg/kg) was administered via intraperitoneal (ip) injection for 7 consecutive days [22]. In the preventative treatment group, mice were pretreated with nicotine (ip) 1 week prior to MPTP modeling and then treated with nicotine continuously for 1 week. In addition, therapeutic treatment meant that the mice were administered nicotine (ip) on the first day after the MPTP injection. MPTP was dissolved in saline according to the manufacturer’s protocol. The concentration was set as 6 mg/ mL and the target dosage for each mouse was 30 mg/kg [22]. Nicotine was dissolved in DMSO to form the concentration of 20 mg/100 μL. Then the solution was diluted to 10 mL using saline to 2 mg/mL. This was regarded as the primary nicotine solution and was distributed and stored at − 20 °C. Part of this solution was thawed and diluted to 0.2 mg/mL and 0.02 mg/mL for in vivo experiments. The administration method was intraperitoneal administration as referenced to Liu et al [23]. The volume was determined by the weight of mice before administration. The weight of each mouse were recorded before intervention every day. As to the vehicle administration, similar dilution strategy was employed just without these reagents. And in the morning, nicotine or corresponding vehicle was administrated. In the afternoon, about 6 h later, MPTP was given as described above. Mice were restricted to food and water on the seventh day of the PD modeling. During that time, we only provided basic food (pellets) and water to maintain daily energy consumption. The pellets were intended to be used as incentives in behavioral tests for olfactory function. On the 8th and 9th days, we trained the mice for behavioral tests three times, and the final tests were performed on the 10th day, including the open field, rotarod, and pole-climbing tests for motor function assessment. Pellet-finding experiments and social discrimination tests were also conducted to assess olfactory function in the different groups. Moreover, nicotine positive group was also added to further explore the benefits of nicotine. Thirty-two mice were randomly allocated to Vehicle, Vehicle + Nic_L, MPTP + Nic_L, and MPTP + Vehicle group (n = 8 in each group). And similar intervention strategies mentioned above were employed to treat these mice. Two independent investigators blinded to the group design conducted the behavioral evaluations. All experiments were conducted in accordance with the guidelines of the Institutional Animal Care and Use Committee of the First Affiliated Hospital of Anhui Medical University (No. PJ2023-09-51).

### Open field test

The open field test is generally employed to evaluate motor activity and exploration intention in rodents [24]. Mice were placed one by one in the center of the open field arena and allowed to search the environment for a specific period of 300 s. During the test, the animal’s behavior was recorded either manually by an observer or automatically using video tracking software. The total travel distance, central travel distance, and static time were calculated. The trajectory route and heatmap were photographed using an animal behavior analysis system developed by Xinruan Corporation (Shanghai, China).

### Rotarod test

The rotarod test provides a measure of motor function and coordination in rodents and is commonly used to evaluate cerebellar function, drug effects, and the impact of neurodegenerative conditions on motor abilities [25]. Mice were trained on the rotarod before the actual test sessions. During training, the rotational speed of the rod was typically constant, and the animals were subjected to multiple trials to improve their motor coordination and balance. Once the animals reached a stable performance level during training, actual test sessions were conducted, often at varying speeds or accelerating rotation (4–40 rpm) for 300 s. The latency was recorded when the mouse fell off the rod. Each mouse was tested and recorded three times. The average time latency for each mouse was calculated.

### Pole-climbing test

The pole-climbing test is a behavioral test commonly used in rodent research to evaluate motor coordination, balance, and motor planning [26]. A vertical rough-surfaced rod with a height of 50 cm was prepared. The mouse was placed on top of the rod, typically with its head facing upward. After training for consecutive 2 days, the performance remained stable, and formal experiments were conducted. The latency from the head turning to the front paws touching the ground was recorded. During these behavior tests, it is necessary to avoid physical exhaustion. Therefore, these three exercise experiments were conducted in three days. After each exercise test, these mice will take a rest for 2–4 h for physical recovery.

### Buried pellets test

To evaluate the odor detection function, a buried pellet test was conducted, as previously described [23, 27]. Briefly, basic food (pellets) and water were provided during the food and water restriction period. During this time, the mice were allowed to familiarize themselves with the pellet. In this experiment, mice tended to have an incentive to find buried pellets. The pellet was randomly buried 0.5–1 cm below the padding at the corner of the test cage (45 × 24 × 20 cm). The mouse was initially placed at the center of the cage. The latency to find the pellet and start eating was recorded. The total duration of the experiment was 5 min. If the mouse did not find the pellet within 5 min, the time was recorded as 5 min. Between different trials, the padding was changed to avoid scent disturbances between the mice.

### Social scent discrimination experiment

The social scent discrimination trial was designed based on previous studies [23, 28]. Briefly, cubic wood (2 × 2 × 2 cm) was incubated with 20 g of mouse padding from the tested mice for 24 h. Mice were habituated to their cages for 1 h without water or food. The mouse was then tested in a place with two wooden cubes (one with its own smell and the other with an unfamiliar smell). Sniffing behaviors were recorded, and the ratio of time spent on each wood cube was calculated and analyzed.

After behavioral evaluation, half of olfactory tissues of three mice in each group were sent for RNA sequencing. The other half of these olfactory tissues were used for RT-qPCR. The half of olfactory tissue of another three mice were employed for Western blot. The other half of these three mice were applied for IHC and IF staining. The remaining two brain tissues were stored in − 80 °C for additional use.

### Transcriptional RNA sequencing

The tissues of mice OBs were collected, immersed in RNAlater RNA Stabilization Reagent (Qiagen, 76,104), and stored at − 80 °C. Transcriptional RNA sequencing was performed by the LC-bio Corporation (Hangzhou, China).

### SYBR Green reverse transcription-quantitative polymerase chain reaction (RT-qPCR)

Total RNA was isolated from the OB using the SparkZol Reagent (AC0101), according to the instructions of manufacturer. The corresponding cDNA library was obtained using a SPARKscript II RT Plus Kit (AG0304) for reverse transcription. Real-time qPCR was conducted employing 2XSYBR Green qPCR Mix (AH0104). GAPDH was used as a reference to normalize prok2R expression. The quantitative analysis was performed using the 2^−△△Ct^ method. The primers were provided by Sangon Biotech. These primers include the following sequences: prok2R: forward, 5′-TACCAACCTCCTCATTGCTAACC-3′; reverse, 5′-GTGGTTTCAAAGGGTGGACAATAG-3′; GAPDH: forward, 5′-CAGTGGCAAAGTGGAGATTGTTG-3′; reverse, 5′-TCGCTCCTGGAAGATGGTGAT-3′.

### Western blot (WB) assay

Treated cells and brain tissues were harvested and stored at − 80 °C. Then RIPA lysis buffer supplemented with fresh protease inhibitor cocktail, phenylmethanesulfonyl fluoride, and phosphatase inhibitor cocktail was used to lyse the cells or brain tissues. The lysates were sonicated using an ultrasonic cell breaker. After incubation on ice for 30 min, the samples were centrifuged at 12,000 rpm for 30 min. The supernatants were collected and quantified using a bicinchoninic acid assay kit (P0012). A loading buffer was added, and the mixture was boiled in a metal bath for further use. Sodium dodecyl sulfate polyacrylamide gel electrophoresis was used to separate the proteins. The extracted protein samples were added into each well, condensed in a 4% stacking gel, and separated in an 8–12% resolving gel under appropriate electric fields. After electrophoresis, the proteins were transferred (blotted) from the gel onto a polyvinylidene fluoride membrane. To reduce the nonspecific binding of antibodies and decrease background noise, the membrane was blocked with a blocking solution containing bovine serum albumin (BSA). A specific primary antibody that recognized the target protein of interest was added to incubate with the membrane overnight at 4 °C with gentle shaking. During this process, the primary antibody binds to the target protein to form an antigen–antibody complex. The TBS-T was used to wash the membrane three times to remove any unbound or nonspecifically bound antibodies. The corresponding secondary antibody was then added and incubated with the membrane to form a sandwich-like structure around the target protein. Finally, the Tanon 5200 system (Tanon Technology Co., Ltd.) was used to capture protein band signals from the membrane.

### Immunohistochemical (IHC) and Immunofluorescence (IF) staining

IHC staining was performed as previously described [29]. 10% neutral-buffered formalin was employed to fix the samples for 24 h. Samples were embedded in paraffin, and standard coronal brain sections (40 μm) were sliced. Paraffin-embedded tissue sections were deparaffinized by heating and treated with xylene to remove wax. The sections were rehydrated using a series of graded alcohol washes (anhydrous, 85%, and 75% ethanol). The citrate buffer (10 mM sodium citrate, pH 8.5) at 90 °C for 30 min was employed to retrieve antigen. PBS was used to wash the sections for three times. The blocking buffer containing 3% BSA, 0.1% Triton X-100, and 0.05% Tween 20 in PBS was used to permeablize these sections. Primary antibodies against TH (rabbit pAb, 1:500) and Cl-casp3 (rabbit mAb, 1:500) were incubated with the slices overnight at 4 °C. After three times of washes with PBS, the Alexa dye-conjugated secondary antibodies (for IHC) or FITC/Cy3-labeled secondary antibodies (for IF) were added to incubate with these sections for 1 h around 25 °C. Hoechst stain (for IHC) or antifade mounting medium with DAPI (for IF) was added to the sections and incubated for 5–10 min at 25 °C to label the nuclei. The slices were evaluated under the XYZ motorized stage connected with Olympus OX41 microscope and counted by a researcher who was blinded to the grouping information using the Optical Fractionator probe of Stereo Investigator software [30, 31]. The quantified striatal TH-positive terminal density was analyzed by densitometry using Image-J software.

### In vitro experiments

Human embryonic kidney 293 T (HEK293T) cell line and primary mouse olfactory neurons (CP-M145) were purchased from Procell (Wuhan, China). HEK293T cells were cultured in Dulbecco’s modified Eagle’s medium supplemented with 10% fetal bovine serum and 1% penicillin–streptomycin solution in an incubator (pO_2_, 21%; 5% CO_2_). Primary mouse olfactory neurons were incubated in the specific medium provided by the manufacturer.

### Cell viability experiment

A Cell Counting Kit-8 (CCK8) assay was used to assess the cell viability. A 96-well plate was seeded with 5000 cells per well and cultured for 24 h to a cell confluence of approximately 60–70% per well. Subsequently, gradient concentrations of nicotine and MPP^+^ were added. After incubation for 24 h, CCK8 solution (10 μL) was added into each well and incubated for 2 h. Finally, a microplate reader was used to record the at 450 nm.

### Live/dead staining

HEK293T cells were seeded in 6-well plated and incubated for 24 h. We then employed different treatment strategies and classified them into several groups: group 1, normal control; group 2, nicotine treatment group; group 3, nicotine + MPP^+^ treatment group (nicotine was added 4 h before MPP^+^ treatment); and group 4, MPP^+^ treatment group. After incubation for 24 h, the cells were washed and incubated with calcein AM and propidium iodide for 30 min. The cells were then gently rinsed three times with PBS to remove the dye. Afterward, these treatment groups were photographed using an inverted fluorescence microscope (ECLIPSE Ti2; Nikon, Japan).

### Analysis of apoptosis using flow cytometry

The cells in the different groups were collected, washed, and resuspended in the medium provided in the Annexin-V and PI Apoptotic Assay Kit. The cells were then stained with annexin-V and propidium iodide solutions. CytoFlex (Beckman Coulter) was used to calculate and analyze the number of apoptotic cells in different groups.

### Transmission electron microscope (TEM) analysis

Cells from the different groups were collected and fixed using 2.5% glutaraldehyde. The fixed samples were dehydrated using a series of alcohol or acetone washes to remove water and prevent electron-beam scattering during imaging. The dehydrated samples were infiltrated with an epoxy resin to provide structural support and facilitate thin sectioning. The resin was then polymerized to form a solid block. Ultrathin sections (70–90 nm thick) were cut from the embedded blocks using an ultramicrotome. Sections were mounted on a TEM copper grid. After staining with uranyl acetate and lead citrate, TEM imaging was performed using Hitachi-7800.

### Construction and transfection of lentivirus

Lentiviruses containing prok2R shRNA and non-silencing control shRNA (shNS) were constructed and provided by GeneChem (Shanghai, China). Similarly, prok2R overexpression (prok2R^+^) and negative control (prok2R^vector^) lentiviruses were also produced. The shRNA target sequences were designed as follows: shNS, 5′-TTCTCCGAACGTCACGT-3′; prok2R-RNAi (shProk2R#1), 5′-GCTGAGACCTATAACCCTGAT-3′; prok2R-RNAi (shProk2R#2), 5′-GCAGATTTAATAGACGAGTAT-3′; prok2R-RNAi (shProk2R#3), 5′-GAAAGGATAGTCAAAGCTGAT-3′. HEK293T cells were transfected according to the manufacturer’s instructions. Then the infected cells were incubated with puromycin (2.0 µg/mL) to screen the successfully transfected cell lines. Finally, the WB was used to evaluate the silencing efficiency of the target genes.

### Confocal laser scanning microscopy (CLSM) analysis of cells

HEK293T cells or primary olfactory neuron-climbing sheets were cultured for 24 h. The sheets were then washed, fixed, and permeabilized. We used 3% BSA to block the antigen for 1 h. Then, the primary prok2R, OMP, and Cl-casp3 antibodies were incubated with the sheets overnight at 4 °C. The sheets were washed with PBS and incubated with FITC/Cy3-labeled secondary antibodies for 1 h. The sheets were then observed, and images were captured using Zeiss LSM880.

### Statistical analysis

The SPSS software (version 19.0; IBM, Armonk, NY, USA) and GraphPad Prism (8.0.2) software were employed to calculate statistical analyses. Data were presented as the mean ± standard deviation, unless indicated otherwise. Data distribution normality was determined by Shapiro–Wilk test. If the data distribution was normalized distributed, Student’s t-test and one-way analysis of variancewere used to evaluate continuous data comparing the differences between two groups and across multiple groups, respectively. Otherwise, Kruskal–Wallis test could be adopted to conduct further analysis. Bonferroni corrections were employed to control type I error for normalized distributed data. False discovery rate (FDR) comparison corrections were applied to reduce type I error for non-normalized distributed data. The two-sided adjusted p-value less than 0.05 was considered significant (*p < 0.05, **p < 0.01, ***p < 0.001).

## Results

### Nicotine alleviated olfactory deficits and motor dysfunctions in MPTP PD mice

**Fig. 1:**
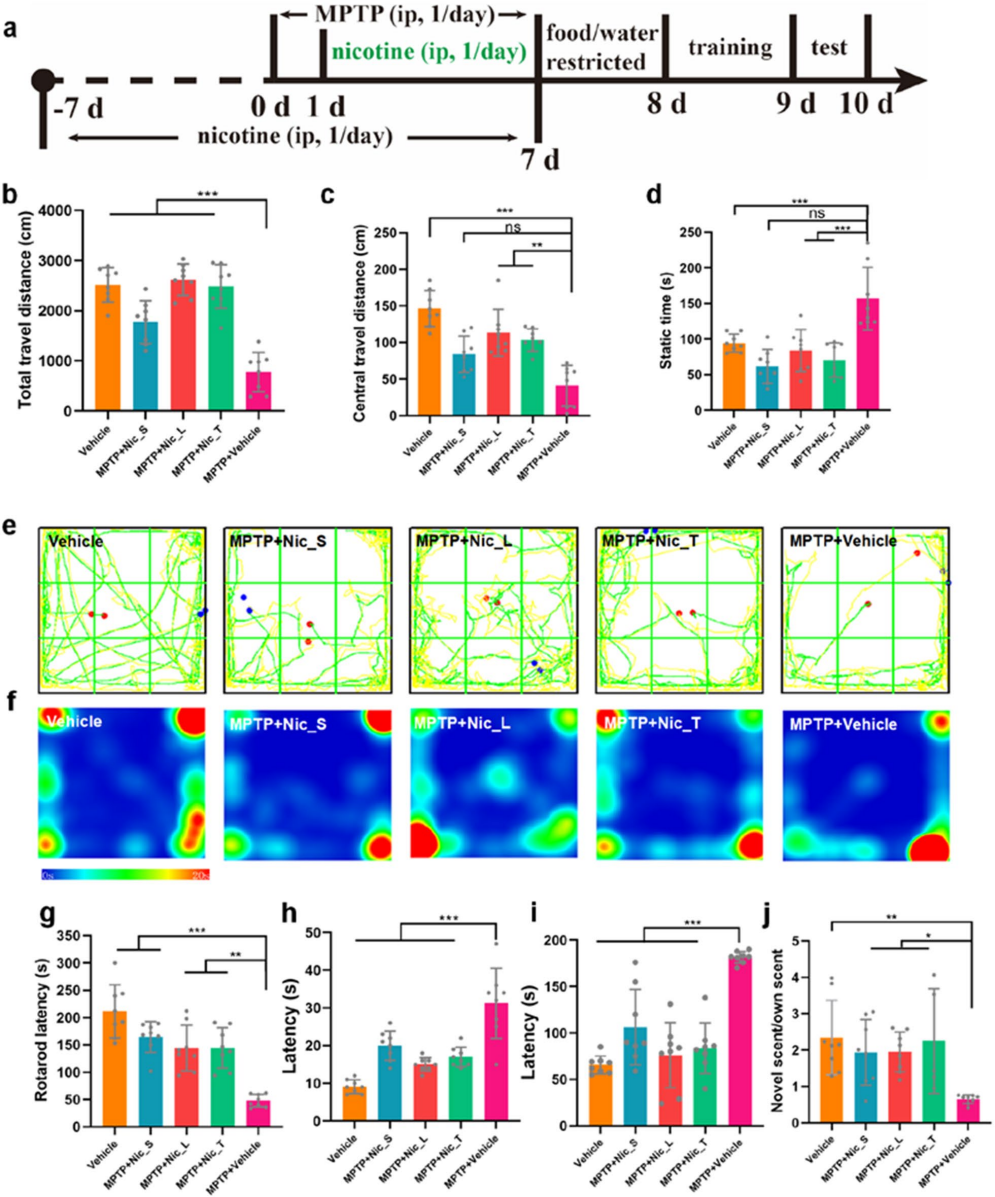
Nicotine alleviates the motor and olfactory performances of MPTP PD mice. **a** Schematic design of in vivo experiments. Total travel distance (**b**), central travel distance (**c**), static time (**d**), route trajectory (**e**), and heatmap (**f**) of the open field test. **g** Rotarod latency in the rotarod test. (h) Latency from the head turning to the front paws approaching the ground. **i** Time latency to find the pellet. (j) The ratio of time spent smelling novel scent versus own scent. Significant differences are shown by *p < 0.05, **p < 0.01, ***p < 0.001. n = 8 in each group

Six-week-old male C57BL/6 mice were randomly allocated into five groups (eight mice per group) with various treatment paradigms: normal control group injected with vehicle (Vehicle), small dosage preventative nicotine group (MPTP + Nic_S), large dosage preventative nicotine group (MPTP + Nic_L), large dosage therapeutic group (MPTP + Nic_T), and MPTP PD model group (MPTP + Vehicle) **(**Fig. 1a**)**. To evaluate the motor function, open field, rotarod, and pole-climbing tests were conducted. The total travel distances of total (Fig. 1b) and central (Fig. 1c) areas were significantly longer in the MPTP + Nic_L and MPTP + Nic_T groups than in the MPTP + Vehicle group. The static time in the MPTP + Nic_L and MPTP + Nic_T groups was significantly decreased compared with that in the MPTP + Vehicle group (Fig. 1d). The trajectory route plot (Fig. 1e) and heatmap plot (Fig. 1f) revealed by the open field test indicated that mice in the MPTP + Nic_S, MPTP + Nic_L, and MPTP + Nic_T groups tended to be more curious about the unfamiliar environment and explored more than mice in the MPTP + Vehicle group. The rotarod test indicated that the latency was significantly longer in the MPTP + Nic_S, MPTP + Nic_L, and MPTP + Nic_T groups than in the MPTP + Vehicle group (Fig. 1g). Moreover, the time from the head turning to the front paws approaching the land was significantly shorter in the MPTP + Nic_S, MPTP + Nic_L, and MPTP + Nic_T groups than in the MPTP + Vehicle group, implying a motor restoration effect of nicotine in the MPTP PD mice (Fig. 1h). Olfactory function was evaluated using olfactory behavioral tests. The mice in the MPTP + Nic_S, MPTP + Nic_L, and MPTP + Nic_T groups spent less time finding pellets than the MPTP PD mice (Fig. 1i). Subsequently, a social discrimination test was performed. The time to smell the novel cubic wood tended to be significantly longer in the MPTP + Nic_S, MPTP + Nic_L, and MPTP + Nic_T groups than in the MPTP + Vehicle group, indicating an improvement in the social smell discrimination capability after nicotine treatment (Fig. 1j). Weight was monitored every 2 days, as shown in Additional file 1: Fig. S1.

### Nicotine partially preserved DA functions in MPTP PD mice

Nissl and IHC staining of TH were performed to estimate the number of remaining DA neurons in MPTP-induced PD mice after nicotine treatment. As depicted in Additional file 2: Fig. S2a and S2b, the number of Nissl^+^ neurons were significantly higher in the preventative group (MPTP + Nic_L) and the therapeutic group (MPTP + Nic_T) than in the MPTP + Vehicle group. TH^+^ neurons in the SNpc (Additional file 2: Fig. S2c and S2d) and TH^+^ fibers in the striatum (Additional file 2: Fig. S2e and S2f) showed similar alterations. Finally, WB results using protein extracted from midbrain area also revealed that TH expression was significantly high in MPTP-induced PD mice treated with nicotine (Additional file 2: Fig. S2g and S2h). These results indicated that DA neurons can be protected by nicotine, thereby improving motor symptoms.

### Prok2R/Akt/FoxO3a axis was activated to prevent apoptosis of olfactory sensory neurons in vivo

**Fig. 2:**
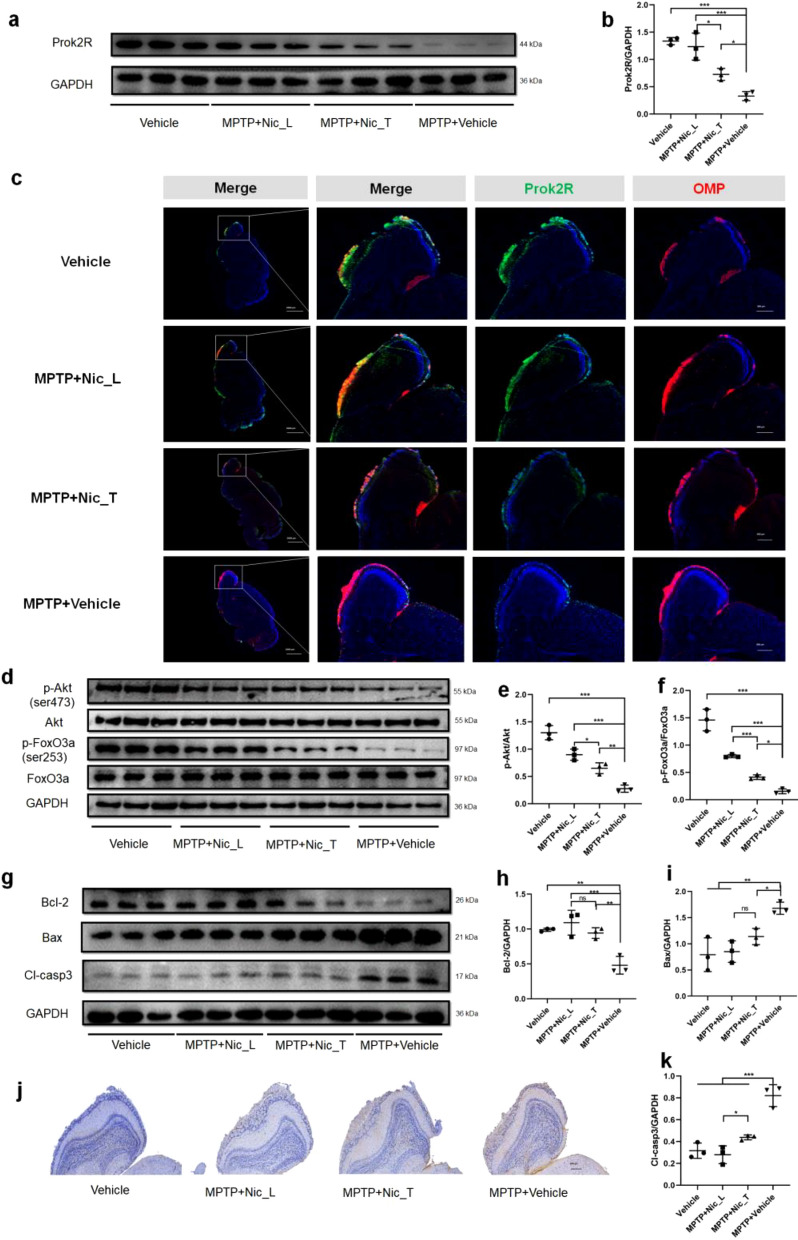
Prok2R expression is recovered by nicotine treatment, and subsequent Akt/FoxO3A axis activity is maintained, thus mitigating the apoptosis of olfactory sensory neurons. Expression levels of prok2R evaluated by western blot (WB) (**a**, **b**). Three immunofluorescence staining images of the olfactory bulb in the sagittal slice of the mouse brain indicate that prok2R is mainly expressed in the glomerular layer cells (**c**). Scale bar = 500 μm. WB results of the Akt/FoxO3a axis in different treatment groups (**d**–**f**). WB images of apoptosis-related proteins, including Bcl-2, Bax, and Cl-casp3 (**g**–**i**). Immunohistochemical staining of Cl-casp3 in sagittal slices of the olfactory bulb (**j**, **k**). Scale bar = 200 μm. Significant differences are shown by *p < 0.05, **p < 0.01, ***p < 0.001. n = 3 in each group

We performed RNA sequencing of the olfactory bulb in different treatment groups (Additional file 3: Fig. S3). A log2FC value of 1.0 and an FDR value of < 0.05 were set as thresholds to define significantly differentially expressed genes (DEGs) (Additional file 3: Fig. S3a, Table S1). A comparison between the MPTP + Vehicle and Vehicle groups revealed 67 DEGs (Additional file 3: Fig. S3a, S3b, S3d, and Additional file 11: Tables S2-S4). Similarly, when comparing the MPTP + Nic_T and MPTP + Vehicle groups, 26 DEGs were found (Additional file 3: Fig. S3a, S3c, S3e, and Additional file 11: Tables S5–S7). We identified 12 common genes between the two DEG cohorts. Among these, prok2R was one of the six significantly downregulated DEGs in the MPTP + Vehicle group compared with the Vehicle group, whereas it was upregulated in the MPTP + Nic_T group compared with the MPTP + Vehicle group (Additional file 3: Fig. S3f, Table 1). To further validate the prok2R level in different treatment groups, WB and RT-qPCR experiments were performed. The expression levels of prok2R were significantly higher in the preventative and therapeutic groups than that in the MPTP + Vehicle group (Fig. 2a, b, Additional file 4: Fig. S4). The expression of prok2 was also evaluated using WB, which revealed that prok2 was not significantly altered among different intervention groups in our study (Additional file 5: Fig. S5). IF staining showed that prok2R expression partially recovered after nicotine treatment. Furthermore, the expression of prok2R was mainly observed in the glomerular layer of the OB, where olfactory sensory neurons synapse with the secondary neurons (mitral/tufted cells). Co-staining of olfactory sensory neurons marked with the olfactory marker protein (OMP) revealed that prok2R was mainly expressed in olfactory sensory neurons (Fig. 2c). WB results indicated that the expression levels of p-Akt (ser473) and p-FoxO3a (ser253) were significantly higher in the Vehicle, MPTP + Nic_L, and MPTP + Nic_T groups than in the MPTP + Vehicle group (Fig. 2d–f). The pro-apoptotic protein Bcl-2-associated X protein (Bax) was significantly downregulated, whereas the anti-apoptotic protein B-cell lymphoma 2 (Bcl-2) was significantly upregulated after nicotine delivery compared with the MPTP + Vehicle group (Fig. 2g–i). Moreover, the apoptotic executor protein cleaved caspase-3 (Cl-casp3) was significantly downregulated in the nicotine treatment group, whereas the expression of Cl-casp3 was high in the MPTP + Vehicle group (Fig. 2g, j, and k).

**Table 1:** Differentially expressed genes by veen plot

Gene_names	MPTP_Vehicle VS Vehicle[log2(FC)]	MPTP_Nic_T VS MPTP_Vehicle[log2(FC)]
Igkv10-96	− 15.12	15.45
Lcn2	− 2.51	2.41
Crabp1	− 2.22	2.12
Hif3a	− 1.29	1.31
Prok2r	− 1.23	1.33
Mt2	− 1.06	1.22
Gm16867	3.49	− 3.18
Gm27177	3.53	− 2.37
Gm5859	3.78	− 3.57
Gm17167	3.9	− 3.06
Gm10599	4.22	− 4.2
Gm17081	4.26	− 3.98

Additionally, the nicotine positive group was also added to further explore the prok2R/Akt/FoxO3a signaling pathway. Nicotine significantly improved olfactory function in MPTP PD mice (Additional file 6: Fig. S6a and S6b). Prok2R/Akt/FoxO3a axis was significantly activated by nicotine in Vehicle + Nic_L and MPTP + Nic_L groups as compared to MPTP + Vehicle group (Additional file 6: Fig. S6c, S6d, S6e, and S6f). And Bcl-2 was partially recovered in MPTP + Nic_L group as compared to MPTP PD mice (Additional file 6: Fig. S6c and S6g). Furthermore, Bax and Cl-casp3 were significantly lower in MPTP + Nic_L group than those in MPTP + Vehicle group (Additional file 6: Fig. S6c, S6h, and S6i). In summary, nicotine can significantly activate prok2R/Akt/FoxO3a and mitigate apoptosis of olfactory bulb neurons in in vivo experiments.

### Nicotine promoted the expression of prok2R by activating downstream Akt/FoxO3a, thus alleviating apoptosis in vitro both in cell lines and primary olfactory neurons

**Fig. 3:**
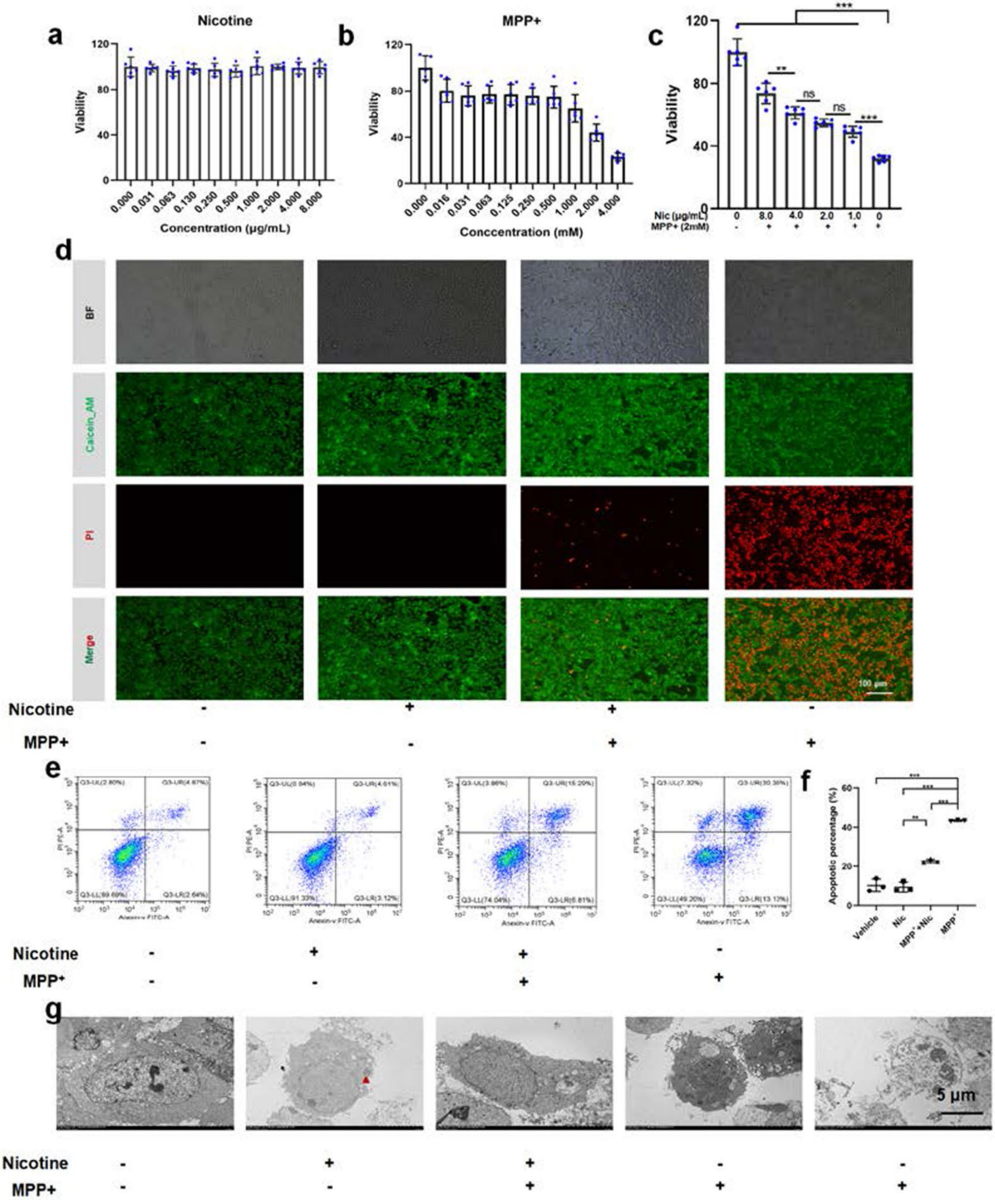
Nicotine treatment mitigates MPP^+^-induced apoptosis in HEK293T cells. **a** Safety evaluation of nicotine using the Cell Counting Kit-8 (CCK 8) assay. n = 6 in each group. **b** Damage assessment of MPP^+^ using the CCK8 assay. n = 6 in each group. **c** Improvement in cell viability owing to nicotine treatment is concentration dependent. n = 6 in each group. **d** The bright field and live/dead staining images of different treatment groups. Scale bar = 100 μm. **e**, **f** Apoptotic flow cytometry and quantitative analyses of the apoptotic cell percentage. n = 3 in each group. **g** Transmission electron microscopy images of HEK293T cells. The phagosome (red triangle labelled) in the nicotine only treated group indicates engulfment of small molecules, and the HEK293T cells of the nicotine + MPP^+^ group are intact. MPP^+^ treated HEK293T cells manifest early apoptosis accompanied by nuclear fragmentation, condensation, and membrane integrity. These cells show ruptured membranes and the disappearance of normal cellular structures during late apoptosis. Scale bar = 5 μm. Significant differences are shown by *p < 0.05, **p < 0.01, ***p < 0.001

HEK293T cell lines are generally used to explore odorant functions [32, 33]. First, the incubation dosage of nicotine was explored to determine appropriate concentrations. Gradient concentrations of nicotine were incubated with HEK293T cell lines for 24 h. The cell viability was estimated by a CCK8 assay. The half-maximal inhibitory concentration (IC50) of nicotine was calculated to be 3.66 mg/mL (Additional file 7: Fig. S7a and S7b). Safety experiments indicated that nicotine did not damage HEK293T cells, even with a concentration of 8 μg/mL (Fig. 3a). Similarly, MPP^+^ was evaluated, and the IC50 was 1.5 mM in HEK293T cells (Fig. 3b, Additional file 7: Fig. S7c). HEK293T cells incubated with nicotine for 4 h were protected from MPP^+^ damage in a concentration-dependent manner (Fig. 3c). The in vitro groups were defined as follows: vehicle only group (Vehicle group), nicotine-only treated group (Nic group), nicotine- and MPP^+^-treated group (MPP^+^ + Nic group), and MPP^+^ + Vehicle PD in vitro model group (MPP^+^ + Vehicle group). The bright field images revealed that the morphology of the cells remained unchanged in the Nic group, whereas shrinkage of the cells and loss of dendrites occurred in the MPP^+^ group (Fig. 3d). Live/dead staining using calcein AM and propidium iodide further proved that nicotine treatment prevented cell death (Fig. 3d). Flow cytometric analysis was performed to evaluate the proportion of apoptotic cells in different groups. Nicotine treatment significantly decreased the proportion of apoptotic cells (Fig. 3e and f). To further confirm subcellular apoptotic alterations in apoptotic cells, TEM was employed. Nicotine treatment prevented apoptosis, as shown in the low-scale TEM images (Additional file 8: Fig. S8). In the Nic group, phagosomes indicated the engulfment of nicotine by cells (red triangle). In contrast, in the MPP^+^ + Nic group, the cells remained normal. In the MPP^+^ + Vehicle group, early and late apoptotic cells were observed. Early apoptotic cells showed nuclear condensation, fragmentation, and loss of mitochondrial integrity, whereas the membrane remained intact. Late apoptotic cells exhibited membrane rupture, formation of apoptotic bodies, and disappearance of normal cellular structures **(**Fig. 3g**)**. Taken together, nicotine effectively prevented MPP^+^-induced apoptosis.

**Fig. 4:**
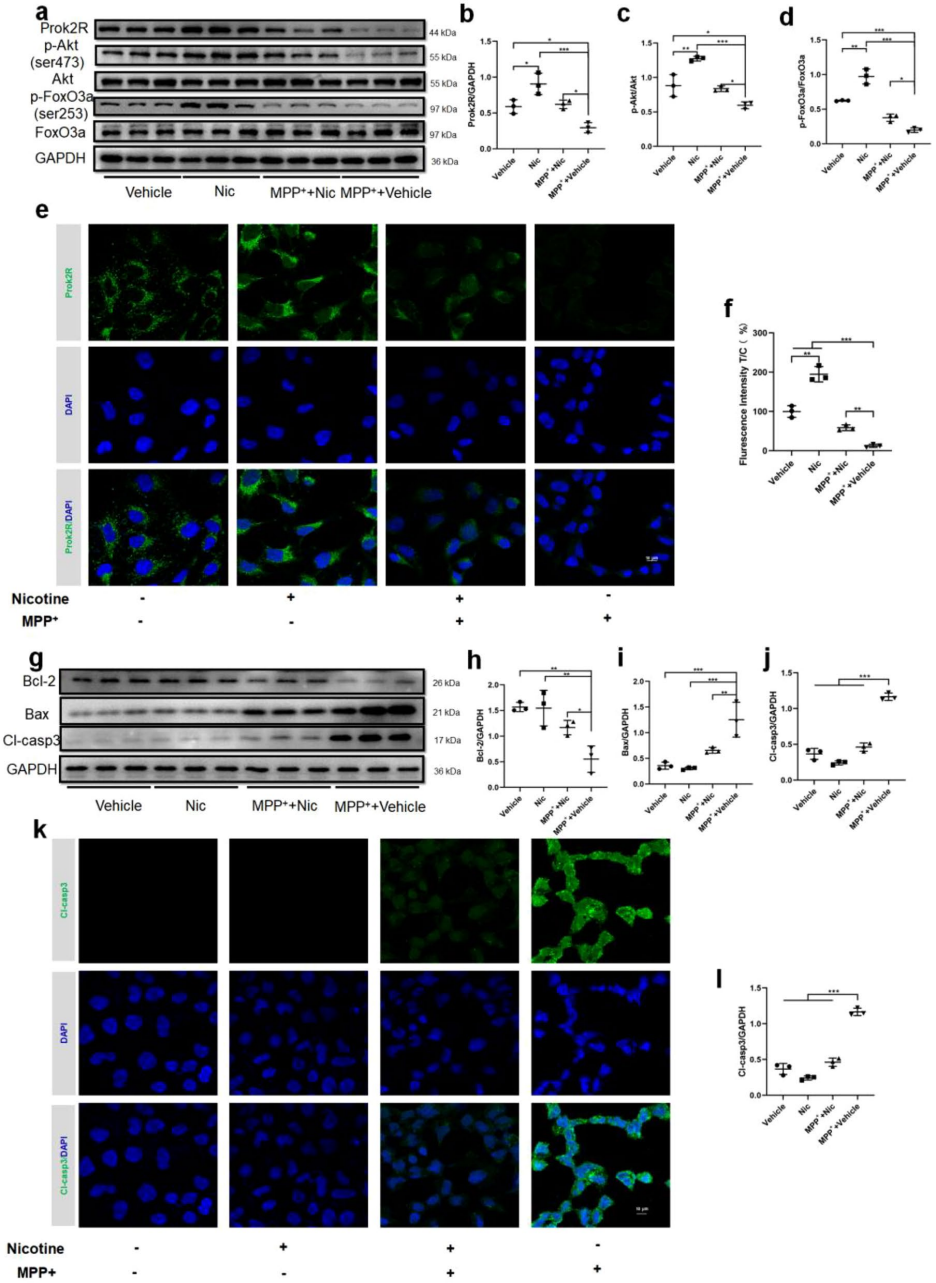
Nicotine activates the prok2R/Akt/FoxO3a axis and exhibits anti-apoptotic effects against MPP^+^ in the HEK293T cell line. Western blot (WB) images (**a**) and semi-quantitative analysis (**b**–**d**) of prok2R/Akt/FoxO3a axis. Confocal laser scanning microscopy (CLSM) images of fluorescein isothiocyanate (FITC)-labeled prok2R (**e**) and semi-quantitative analysis of fluorescence intensity (**f**). Scale bar = 10 μm. The expression levels of anti-apoptotic protein (Bcl-2), pro-apoptotic protein (Bax), and apoptotic executor protein (Cl-casp3) detected using WB (**g**–**j**). **k**, **l** CLSM of FITC-labeled Cl-casp3 indicating decreased expression levels after nicotine treatment against MPP^+^ damage. Scale bar = 10 μm. Significant differences are shown by *p < 0.05, **p < 0.01, ***p < 0.001. n = 3 in each group

In the signaling pathway analysis, we found that elevated prok2R expression activated the expression of p-Akt and p-FoxO3a, which were significantly upregulated in the Nic and MPP^+^ + Nic groups compared with the MPP^+^ + Vehicle group (Fig. 4a–d). The prok2 protein was not significantly expressed in these groups (Additional file 9: Fig. S9). CLSM also indicated that prok2R expression was mainly located in the membrane and was upregulated after nicotine treatment against MPP^+^-induced reduction (Fig. 4e and f). The levels of apoptosis-related proteins were also assessed. Bcl-2 expression significantly recovered after incubation with nicotine (Fig. 4g and h) in the MPP^+^ + Vehicle group. Bax expression was significantly downregulated following nicotine treatment (Fig. 4g and i). Consequently, Cl-casp3 was inhibited in the MPP^+^ + Nic group (Fig. 4g and j), indicating a decrease in apoptosis. CLSM confirmed these alterations in Cl-casp3 in the different treatment groups (Fig. 4k and l). Collectively, nicotine significantly activated the prok2R/Akt/FoxO3a axis to prevent apoptosis in HEK293T cells.

**Fig. 5:**
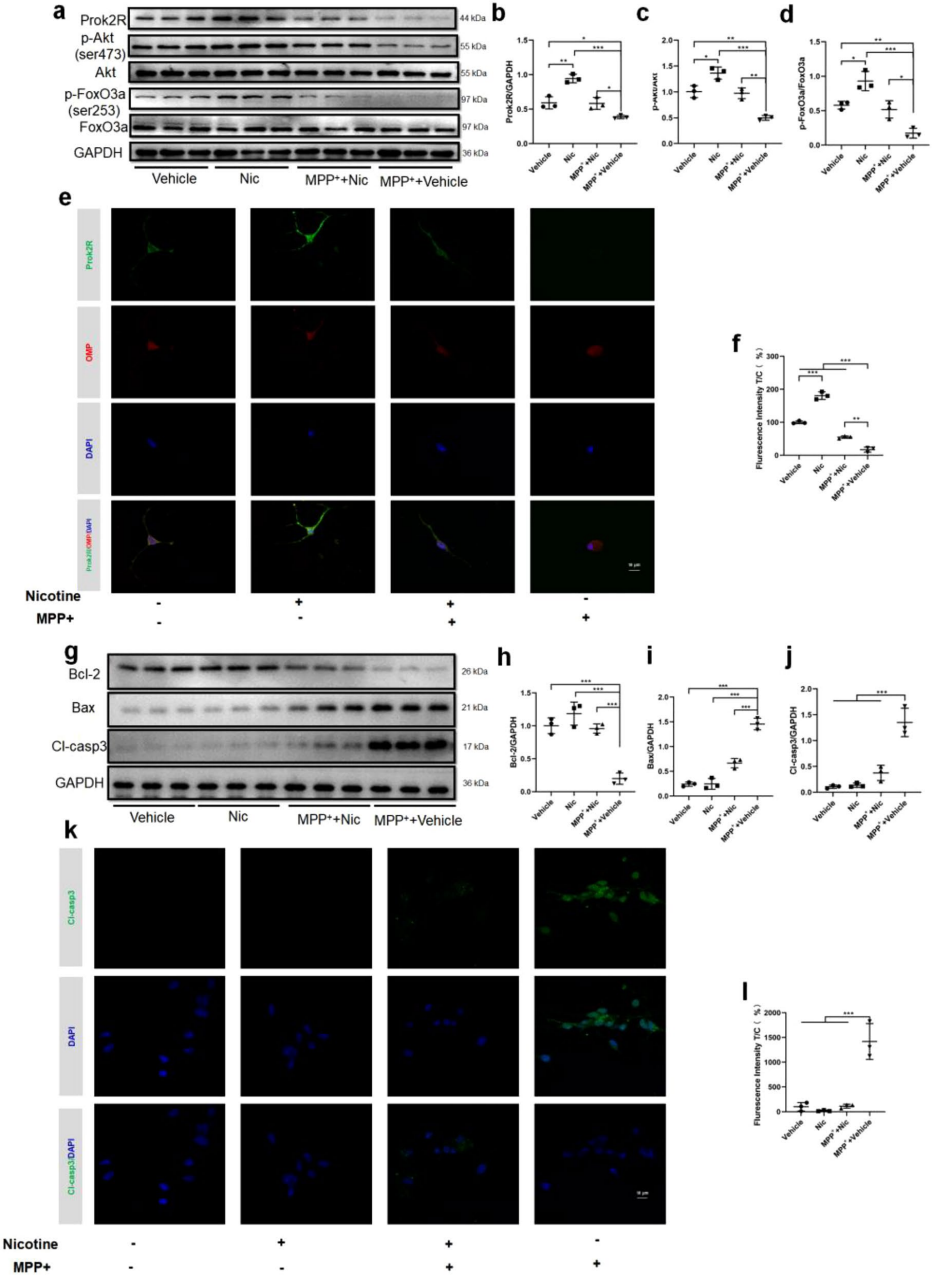
Nicotine activates the prok2R/Akt/FoxO3a axis and exhibits anti-apoptotic effects against MPP^+^ in the primary olfactory sensory neurons. Western blot (WB) images (**a**) and semi-quantitative analysis (**b**–**d**) of prok2R/Akt/FoxO3a axis. Confocal laser scanning microscopy (CLSM) images of fluorescein isothiocyanate (FITC)-labeled prok2R (e) and semi-quantitative analysis of fluorescence intensity (**f**). Scale bar = 10 μm. The expression levels of anti-apoptotic protein (Bcl-2), pro-apoptotic protein (Bax), and apoptotic executor protein (Cl-casp3) detected using WB (**g**–**j**). **k** CLSM of FITC-labeled Cl-casp3 indicating decreased expression levels after nicotine treatment against MPP^+^ damage. Scale bar = 10 μm. Significant differences are shown by *p < 0.05, **p < 0.01, ***p < 0.001. n = 3 in each group

Primary olfactory neurons isolated from the mouse OB were used to explore the prok2R/Akt/FoxO3a axis and its corresponding anti-apoptotic effects. The CCK8 assay indicated that nicotine was safe, even at a concentration of 8 μg/mL (Additional file 7: Fig. S7d). Subsequently, prok2R expression was explored in the different intervention groups. After incubation with nicotine, the expression of prok2R significantly increased, which acted as a protective factor by activating downstream p-Akt and p-FoxO3a (Fig. 5a–d). The prok2 protein was not significantly expressed in these groups (Additional file 10: Fig. S10). The CLSM images indicated that prok2R (green) was significantly highly expressed in the Vehicle, Nic, and Nic + MPP^+^ groups with OMP-positive neurons (labeled red) compared with the MPP^+^ + Vehicle group (Fig. 5e and f). Apoptotic proteins were assessed in all the four groups. Bcl-2 expression was significantly elevated in the Vehicle, Nic, and Nic + MPP^+^ groups as compared with the MPP^+^ + Vehicle group (Fig. 5g and h). Bax expression was efficiently downregulated by nicotine treatment (Fig. 5g and i). Cl-casp3 significantly decreased in the Vehicle, Nic, and Nic + MPP^+^ groups, indicating the anti-apoptotic effects of nicotine in the MPP^+^ + Vehicle group (Fig. 5g–l). Nicotine activated the prok2R/Akt/FoxO3a axis, thus protecting both HEK293T cells and olfactory sensory neurons from MPP^+^-induced apoptotic alterations.

### Prok2R expression regulated the activity of the Akt/FoxO3a signaling pathway

**Fig. 6:**
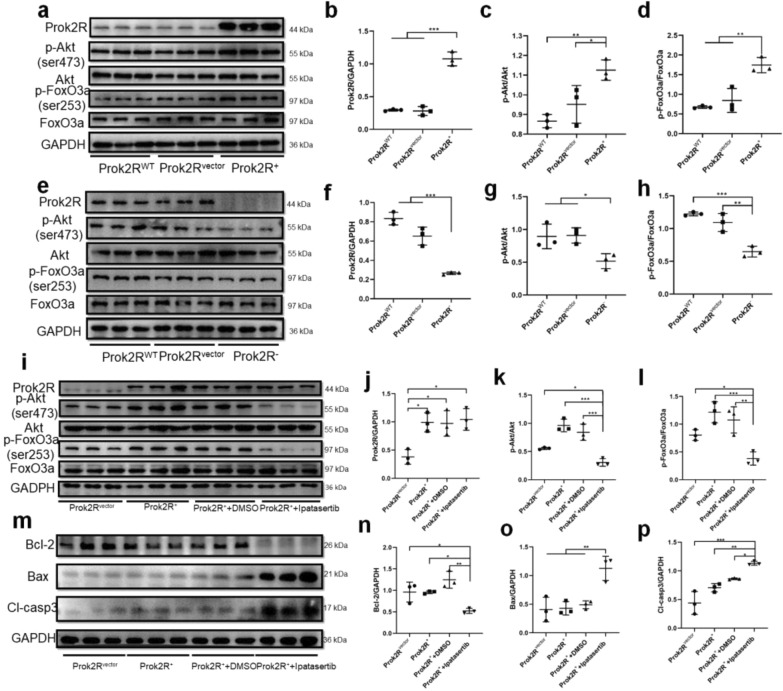
Prok2R expression levels modulate activation of the Akt/FoxO3a axis. Western blot (WB) images of prok2R/Akt/FoxO3a in prok2R^+^ HEK293T cell lines (**a**–**d**). WB images of prok2R/Akt/FoxO3a in prok2R^−^ HEK293T cell lines (**e**–**h**). The prok2R/Akt/FoxO3a axis expression levels in the prok2R^vector^, prok2R^+^, prok2R^+^ + DMSO, and prok2R^+^ + ipatasertib groups (**i**–**l**). Apoptosis-related proteins expressed in different treatment groups (**m**–**p**). Significant differences are shown by *p < 0.05, **p < 0.01, ***p < 0.001. n = 3 in each group

In order to explore the relationship between prok2R and the activation of downstream signaling pathways. Prok2R was overexpressed by transfection with a plasmid containing Ubi-MCS-3FLAG-CBh-gcGFP-IRES-puromycin (the prok2R^+^ group). Simultaneously, the vacant plasmid was successfully transfected with the vector group to be employed as a negative control (prok2R^vector^). The untransfected HEK293T cell line was employed as a normal control (prok2R^WT^), and transfection was proven to be successful (Fig. 6a and b). Moreover, p-Akt and p-FoxO3A were found to be activated during these experiments. However, these effects were not observed in the prok2R^vector^ group (Fig. 6a, c, and d). Then prok2R was knocked down using the plasmid hU6-MCS-CBh-gcGFP-IRES-puromycin (prok2R^−^). A vacant plasmid was constructed as the negative control (prok2R^vector^) (Fig. 6e and f). The untransfected HEK293T cell line was used as the normal control (prok2R^WT^). Consequently, prok2R knockdown deactivated the Akt/FoxO3a axis (Fig. 6e, g, and h).

We further confirmed the participation of the prok2R/Akt/FoxO3a axis in the anti-apoptotic pathway. A novel and ATP-competitive pan-Akt inhibitor, ipatasertib (GDC-0068), was reported to inhibit p-Akt effectively [34]. This inhibitor was employed to reverse the activation of the Akt/FoxO3a axis induced by upregulated prok2R. After incubation with ipatasertib for 12 h, the Akt/FoxO3a signaling pathway was inhibited, despite the upregulation of prok2R (Fig. 6i–l). The levels of apoptosis-related proteins were also assessed. As depicted in Fig. 6m–p, Bcl-2 expression was significantly suppressed after ipatasertib treatment. Bax was highly expressed in the ipatasertib-treated group. Cl-casp3 was significantly higher in the presence of ipatasertib. Therefore, the application of ipatasertib mitigated the anti-apoptotic effect by deactivating the Akt/FoxO3a axis, regardless of the upregulation of prok2R. Taken together, the upregulation of prok2R activated the downstream Akt/FoxO3a signaling pathway, thereby playing a protective role in anti-apoptotic biological processes.

### Upregulation of prok2R protected MPP^+^-induced apoptosis through the preservation of the Akt/FoxO3a activity

**Fig. 7:**
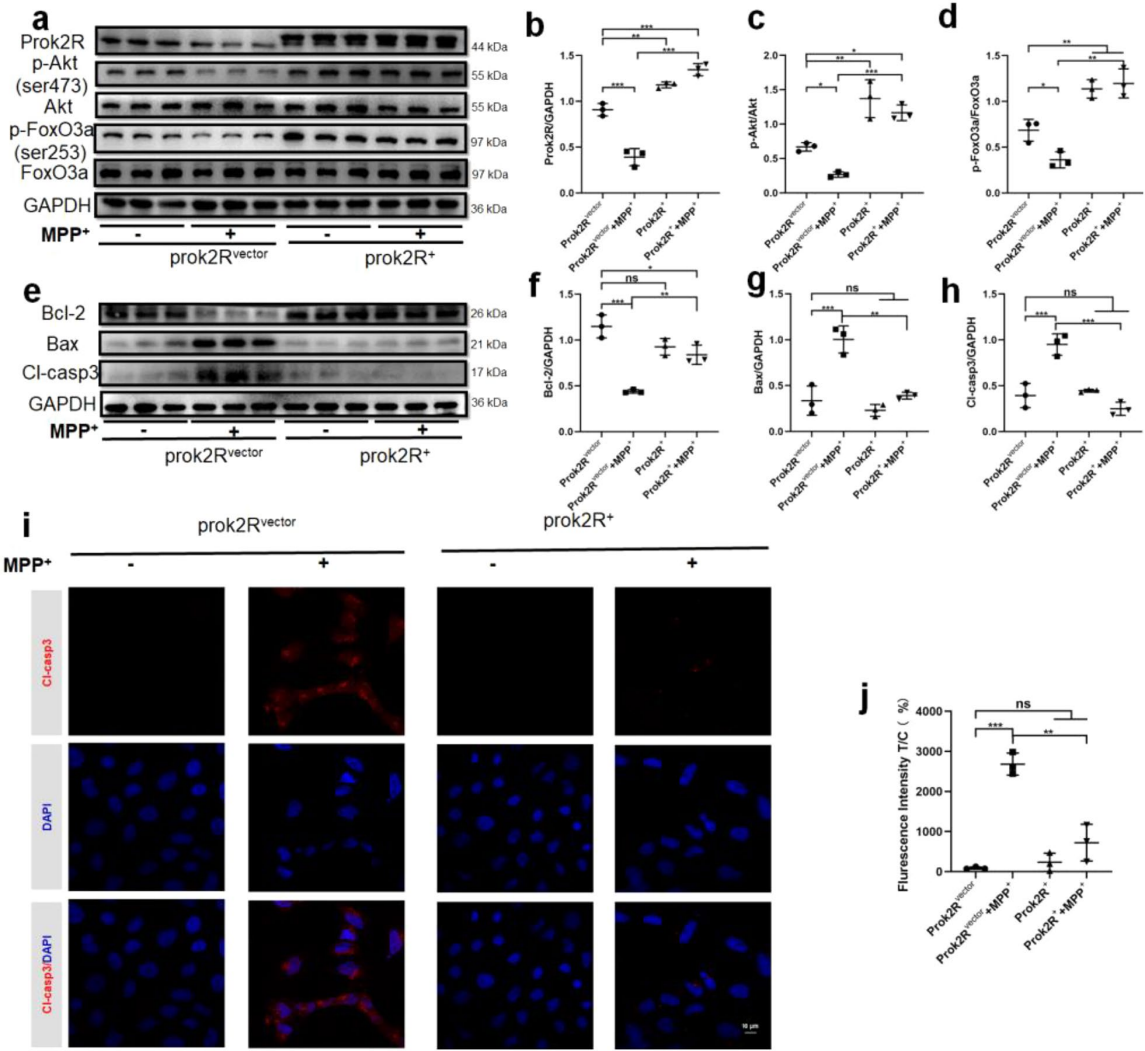
Prok2R^+^ HEK293T cells alleviate MPP^+^-induced apoptosis through the preservation of prok2R/Akt/FoxO3a axis activity. The activity of prok2R/Akt/FoxO3a in different treatment groups (prok2R^vector^, prok2R^vector^ + MPP^+^, prok2R^+^, prok2R^+^ + MPP^+^) revealed using the western blot (WB) assay (**a**–**d**). Apoptosis-related proteins indicated using the WB assay (**e**–**h**). Confocal laser scanning microscopy images of Cy3-labeled Cl-casp3 in each group (**i**, **j**). Scale bar = 10 μm. Significant differences are shown by *p < 0.05, **p < 0.01, ***p < 0.001. n = 3 in each group

Experiments were conducted to explore whether the upregulation of prok2R could alleviate MPP^+^-induced apoptosis. HEK293T cells were divided into four groups: Vector without MPP^+^ (prok2R^vector^), Vector with MPP^+^ (prok2R^vector^ + MPP^+^), prok2R^+^ without MPP^+^ (prok2R^+^), and prok2R^+^ with MPP^+^ (prok2R^+^ + MPP^+^). The expression levels of prok2R, p-Akt, and p-FoxO3a in the prok2R^vector^ + MPP^+^ group were significantly downregulated as compared to the prok2R^vector^ group (Fig. 7a–d). The expression levels of prok2R, p-Akt, and p-FoxO3a were significantly upregulated in prok2R^+^ group and prok2R^+^ + MPP^+^ group as compared to those in the prok2R^vector^ group (Fig. 7a–d). Moreover, the expression levels of prok2R, p-Akt, and p-FoxO3a were significantly higher in prok2R^+^ + MPP^+^ group than those in prok2R^vector^ + MPP^+^ group (Fig. 7a–d). Apoptosis-related proteins were further evaluated, and the results showed a significant downregulation of Bcl-2 in prok2R^vector^ + MPP^+^ group as compared to that in prok2R^vector^ group (Fig. 7e–h). The expression level of Bcl-2 in prok2R^+^ + MPP^+^ group was significantly higher than that in prok2R^vector^ + MPP^+^ group (Fig. 7e–h). Bax and Cl-casp3 were significantly upregulated in prok2R^vector^ + MPP^+^ group as compared to that in prok2R^vector^ group (Fig. 7e–h). The expression levels of Bax and Cl-casp3 in prok2R^+^ + MPP^+^ group were significantly lower than those in prok2R^vector^ + MPP^+^ group (Fig. 7e–h). CLSM indicated that Cy3 labeled Cl-casp3 was significantly upregulated in the prok2R^vector^ + MPP^+^ group as compared to that in the prok2R^vector^ group indicating that the apoptotic alterations were successfully induced by MPP^+^ (Fig. 7i and j). Furthermore, the Cl-casp3 was expressed higher in the prok2R^vector^ + MPP^+^ group than that in the prok2R^+^ + MPP^+^ group (Fig. 7i and j). Thus, all these results indicated that the overexpression of prok2R protected cells from MPP^+^ induced apoptotic alterations by activating Akt/FoxO3a signaling pathway.

## Discussion

**Scheme 1:**
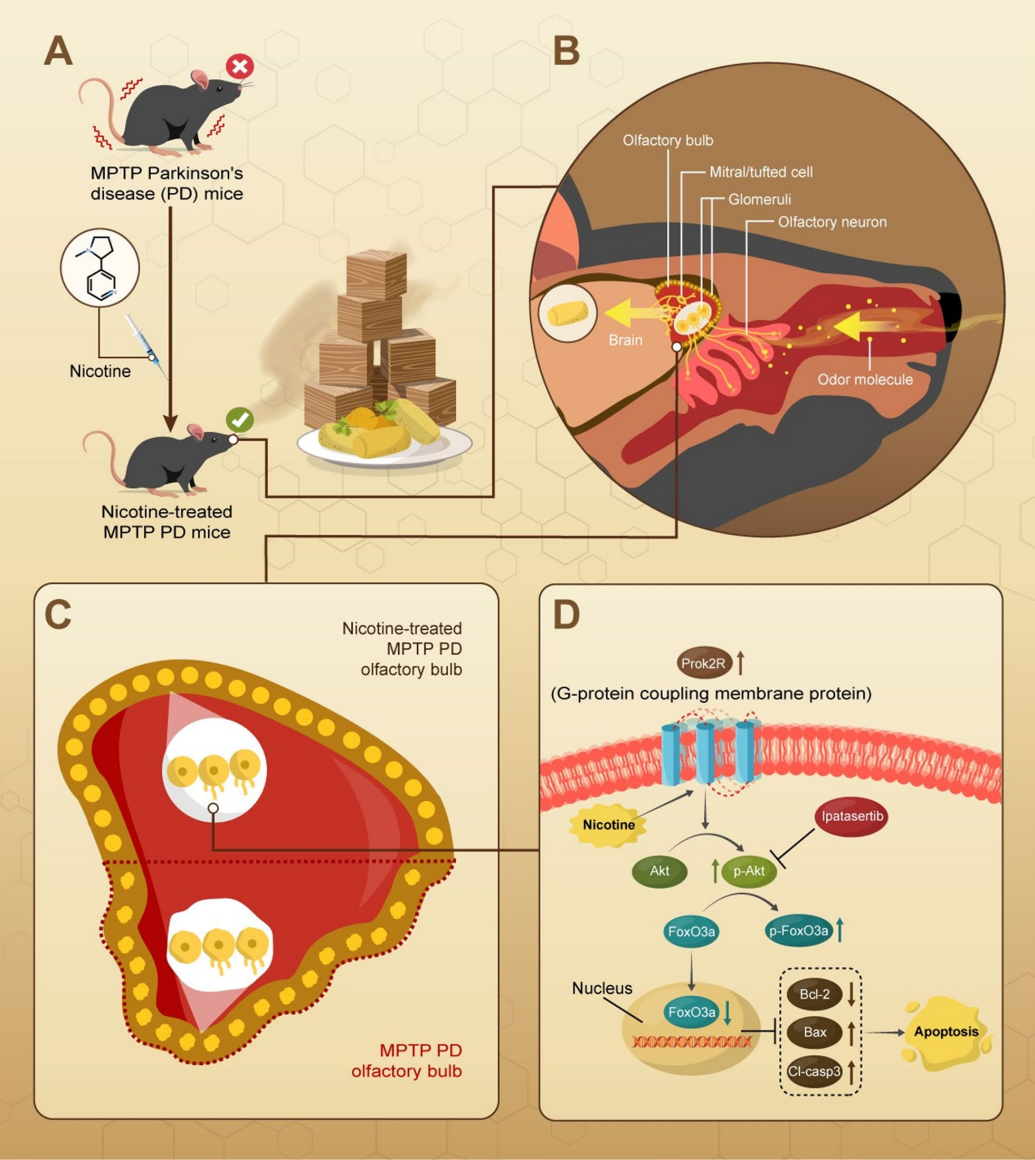
The schematic diagram of this article. The treatment of nicotine can improve the motor and olfactory dysfunctions of MPTP PD mice. This might be related with the activation of prok2R/Akt/FoxO3a axis, thereby mitigating the apoptosis of olfactory sensory neurons in the glomeruli layer of the olfactory bulb. The activation of this pathway can be compromised by the Akt inhibitor ipatasertib

The OB is one of the earliest tissues involved in the pathogenesis of PD, and olfactory dysfunction or hyposmia is commonly found in approximately 90% of patients with PD. Many studies have reported that nicotine ameliorates motor symptoms [10, 35–37]. However, there is a lack of fundamental evidence showing a correlation between olfactory function and PD, although epidemiological and clinical studies have revealed that cigarette smokers tend to have a low risk of PD. This study is the first to explore the mechanisms of nicotine in olfactory deficit mitigation using animal behavioral tests, transcriptional RNA sequencing, and molecular biological methods (Scheme 1).

In behavioral tests, we found that nicotine improved the olfactory and motor performance of MPTP-induced PD mice in both preventative and therapeutic ways. Previous studies reported that large tobacco smoke exposure can damage olfactory neurons, olfactory epithelia, and promote rhinitis symptoms [38–41]. However, for olfactory dysfunction population, current or historic smoking showed no obvious correlation with olfactory loss in sinonasal, postviral, or idiopathic olfactory loss [42]. A systematic meta-analysis indicated that smoking caused a series disease and cancer development regarding circulatory system, digestive system, endocrine system, musculoskeletal system, metabolic system, and eyes. Nevertheless, this article still revealed that smoking was related with decreased PD risks [43]. Similarly, previous epidemiological studies have revealed that the prevalence of PD in smokers was less than that of non-smokers (approximately half) [44, 45]. Therefore, the mechanisms for these phenomena still need further experiments. On the one hand, the elements in tobacco smoking comprise thousands of compounds which are harmful for human body. On the other hand, the dosage of tobacco smoking might also determine whether the effects are protective or harmful. Consequently, figuring out the effective element and appropriate dosage are necessary. Being regarded as the effective element of tobacco, nicotine has been reported to have the capability of preventing apoptosis of DA neurons in PD modeling mice and primates [10, 11]. Meanwhile, nicotine can also decrease the number of activated microglia in the SNpc, implying its anti-inflammatory effects in PD [35]. Furthermore, nicotine activates olfactory sensory neurons by an activation of a canonical olfactory pathway (cAMP-dependent second messenger pathway) instead of nicotinic acetycholine receptors [46]. Yang et al. [23] found that nicotine mitigated hyposmia in acute MPTP-induced PD mice through upregulation of TH and choline acetyltransferase in the OB and horizontal limb of the diagonal band [35]. No underlying mechanism for olfactory function has been explored in depth. Therefore, this article mainly investigated the potential targets and signaling pathway regarding nicotine through different intervention strategies. Our results indicated that nicotine could efficiently mitigate motor and olfactory dysfunctions both at small and large dosages, either in a preventative or a therapeutic way.

This study used transcriptomic RNA sequencing analysis to screen out potential targets in olfactory bulb for nicotine treatment in MPTP PD mice. The results revealed that prok2R was one of the six genes that were most differentially expressed in the nicotine-treated and vehicle-treated PD mice. In the MPTP + Vehicle group, prok2R was significantly downregulated. The treatment of nicotine (MPTP + Nic_T) effectively upregulated prok2R, implying that nicotine could directly regulate the expression of prok2R in the OB tissues of PD mice. As a G protein-coupled receptor, prok2R upregulation activates corresponding downstream signaling pathways and plays a pivotal role in development of olfactory bulb thus influencing olfactory functions. Mutations in prok2R can cause Kallmann syndrome characterized by the combination of hypogonadotropic hypogonadism (delayed or absent puberty) and anosmia [47]. Given the essential role in olfactory development, prok2R might be a novel target in protecting olfactory dysfunction in PD. Previous studies regarding the mechanism of nicotine against PD mainly focused on DA neurons and microglia [35–37, 48]. These studies revealed the mechanisms from multifaceted perspectives, including anti-apoptotic effects either by enhancement of autophagy by the elevated Ca^2+^ influx [37] or the activation of JAK2/STAT3/NF-κB pathway [36]. Other mechanisms (anti-fibrillogenic and anti-oxidative) were also revealed by earlier studies [48, 49]. Yang et al. found that a low dosage of nicotine was capable of restoring the decline of NAMPT activity which was age-related by SIRT1 binding and NAMPT deacetylation, thus resulting in the increased level of NAD^+^ synthesis [50]. Moreover, nicotine was also reported to have the capability of inhibiting SIRT6 and preventing the apoptosis of DA neurons in the MPTP PD mice model [51]. These studies showed that nicotine can stimulate neurogenesis, inhibits neuroinflammation, and protect organs against oxidative stress and telomere shortening. A study reported that smoking targeted human fallopian tubes via nAChR-7 to upregulate tubal prokR1 expression, resulting in alterations in microenvironment which could predispose women to tubal ectopic pregnancies [52]. Furthermore, a clinical study found that prok2 expression was significantly increased in olfactory neurons among patients with PD [12]. The fundamental investigations also proved that elevated prok2 protein reduced neuronal damage in mice with traumatic brain injury and protected DA neurons from MPTP/MPP^+^-induced apoptosis in the early stage of the disease [19]. Matsumoto et al. found that prok2R knockout mice exhibited OB dysplasia, particularly in the glomerular layers where olfactory sensory neurons synapsed with mitral/tufted cells [14]. Meanwhile, the prok2/prok2R pathway was also shown to participate in the neurogenesis of OB [18]. However, the study on the signaling pathway of prok2/prok2R in olfactory dysfunction in PD is still absent. Our results firstly indicated that nicotine might play a neuroprotective role in PD modeling mice, and this effect might be induced by the activation of prok2R-related signaling pathways in the OB.

Prok2R was demonstrated to be positively correlated with its downstream key molecule Akt (ser473), whose activation plays crucial roles in cell survival, proliferation, and apoptosis [19]. FoxO3a, a member of the Forkhead box O family of transcription factors, plays a crucial role in regulating apoptosis, cell cycle arrest, and oxidative stress response [53]. Phosphorylated Akt can directly activate and phosphorylate FoxO3a protein at multiple sites, such as Thr32, Ser253, and Ser315 [53]. This phosphorylation leads to the nuclear exclusion of FoxO3a, preventing its entry into the nucleus and transcriptional activation of its target genes. Some of these target genes are involved in promoting cell cycle arrest and apoptosis, which are crucial for regulating cell survival and maintaining cellular homeostasis [54]. Apoptosis is believed to play a major role in neuronal loss observed in PD. Studies have found increased levels of proteins and enzymes involved in apoptosis in the DA neurons of PD [55]. Several factors may trigger apoptosis, including oxidative stress, mitochondrial dysfunction, inflammation, and problems with protein handling, such as α-synuclein aggregation [56]. Animal models of PD have demonstrated that toxins such as MPTP, rotenone, and 6-hydroxydopamine can induce apoptosis in DA neurons [57, 58]. The Akt/FoxO3a signaling pathway plays an important role in the anti-apoptotic function in PD. Treatments that target this pathway have been investigated as potential therapies to slow neurodegeneration in PD [58–60]. In our previous study, apoptotic cells were found in the glomeruli cell layer in an MPTP-induced cynomolgus monkey model of PD [20]. Furthermore, a three-dimensional construction of the OB postmortem revealed that ventral glomerular layer cells significantly decreased in patients with PD [61]. The apoptosis of olfactory neurons has been reported to be associated with olfactory dysfunction [62]. However, the underlying mechanism was not further explored. Therefore, this study further explored the anti-apoptotic effect and potential mechanism of nicotine in the protection of olfactory sensory neurons and preservation of olfactory function, which have not been previously reported. The expression levels of prok2R were proven to modulate the activity of the Akt/FoxO3a signaling pathway. Furthermore, inhibition of p-Akt using an ATP-competitive p-Akt inhibitor effectively suppressed the activation of Akt/FoxO3a induced by prok2R, thereby leading to apoptosis. Additionally, upregulation of prok2R mitigated MPP^+^-induced apoptosis. Upregulation of prok2R and activation of corresponding downstream Akt/FoxO3a signaling pathway were well elucidated based on above evidences, exerting a protective role against apoptotic pathologic alterations.

## Conclusion

In conclusion, this study successfully screened out elevated prok2R levels in olfactory sensory neurons of the MPTP PD mouse OB after nicotine treatment, using transcriptomic RNA sequencing. The prok2R/Akt/FoxO3a was found to be upregulated both in vivo and in vitro, playing a pivotal role in anti-apoptotic effects in PD models. These findings provide new evidence and perspectives demonstrating the potential of nicotine, related biomarkers, and signaling pathways for the treatment of PD. Nevertheless, future researches are necessary to explore the underlying mechanisms of the upregulation of prok2R and its possible clinical applications.

### Supplementary Information

**Additional file 1: Figure S1.** Weight curves monitored every 2 days during the in vivo experiment. n = 8 in each group.

**Additional file 2: Figure S2.** Dopaminergic neurons are preserved by nicotine treatment both in the preventative and therapeutic manner. Nissl staining (**a**, **b**) and tyrosine hydroxylase (TH) immunohistochemical (IHC) staining (**c**, **d**) of the SNpc in the coronal slice of the mouse brain. TH IHC staining of the striatum (**e**, **f**). Western blot (**g**, **h**) of TH in different treatment groups (Vehicle, MPTP + Nic_L, MPTP + Nic_T, and MPTP + Vehicle). Scale bar = 1000 μm and 400 μm. Significant differences are shown by *p < 0.05, **p < 0.01, ***p < 0.001. n = 3 in each group.

**Additional file 3: Figure S3.** The transcriptional RNA sequence analyses. The DEGs (**a**) screened out by log2FC value 1.0 and FDR value < 0.05. The volcano plot (**b**) and heatmap plot (**d**) of DEGs in MPTP_Vehicle group versus Vehicle group. The volcano plot (**c**) and heatmap plot (**e**) of DEGs in MPTP_Nic group versus MPTP_Vehicle group. Venn plot (**f**) for DEGs in MPTP_Vehicle VS Vehicle and MPTP_Nic_T VS MPTP_Vehicle comparison group.

**Additional file 4: Figure S4.** Expression levels of prok2R evaluated by reverse transcription-quantitative polymerase chain reaction. Significant differences are shown by *p < 0.05, **p < 0.01, ***p < 0.001. n = 3 in each group.

**Additional file 5: Figure S5.** Expression of prok2 revealed using western blot in in *vivo* experiments. n = 3 in each group.

**Additional file 6: Figure S6.** Nicotine can upregulate prok2R/Akt/FoxO3a axis thus protect olfactory from apoptotic alterations thus preserving olfactory functions in MPTP PD mice. Time latency to find the pellet (**a**). The ratio of time spent smelling novel scent versus own scent (**b**). Prok2R (**c**, **d**), p-Akt (**c**, **e**), and p-FoxO3a (**c**, **f**) expression levels evaluated by WB assay. Bcl (c, g), Bax (**c**, **h**), and Cl-caspase-3 (**c**, **i**) levels evaluated by WB assay. Significant differences are shown by *p < 0.05, **p < 0.01, ***p < 0.001. n = 3 in each group.

**Additional file 7: Figure S7.** **a** Viability of HEK293T cells with different doses of nicotine. **b** IC50 calculation curve for nicotine. n = 4 in each group. **c** IC50 calculation curve for MPP + . **d** Safety evaluation experiments of nicotine for primary olfactory bulb neurons. n = 6 in each group.

**Additional file 8: Figure S8.** Transmission electron microscopy images of HEK293T cells from different treatment groups. Scale bar = 20 μm.

**Additional file 9: Figure S9.** Prok2 expression levels in different treatment groups of HEK293T cells assessed using western blot assay. n = 3 in each group.

**Additional file 10: Figure S10.** Prok2 expression levels in different treatment groups of primary olfactory bulb cells assessed using western blot assay. n = 3 in each group.

**Additional file 11: Table S1.** DEGs screened using a log2FC value of 1.0 and an FDR value of < 0.05. **Table S2.** DEGs of the MPTP + Vehicle group versus the Vehicle group. **Table S3.** Significantly downregulated DEGs of the MPTP + Vehicle group versus the Vehicle group. **Table S4.** Significantly upregulated DEGs of the MPTP + Vehicle group versus the Vehicle group. **Table S5.** DEGs of the MPTP + Nic_T group versus the MPTP + Vehicle group. **Table S6.** Significantly downregulated DEGs of the MPTP + Nic_T group versus the MPTP + Vehicle group. **Table S7.** Significantly upregulated DEGs of the MPTP + Nic_T group versus the MPTP + Vehicle group.

## Data Availability

Data will be made available on reasonable request.
