# Supplementary figures and images for: Nicotine restores olfactory function by activation of prok2R/Akt/FoxO3a axis in Parkinson’s disease
Source: J Transl Med. 2024 Apr 12;22:350. doi: 10.1186/s12967-024-05171-1 (PMC11015622; doi:10.1186/s12967-024-05171-1)

## Slide 1
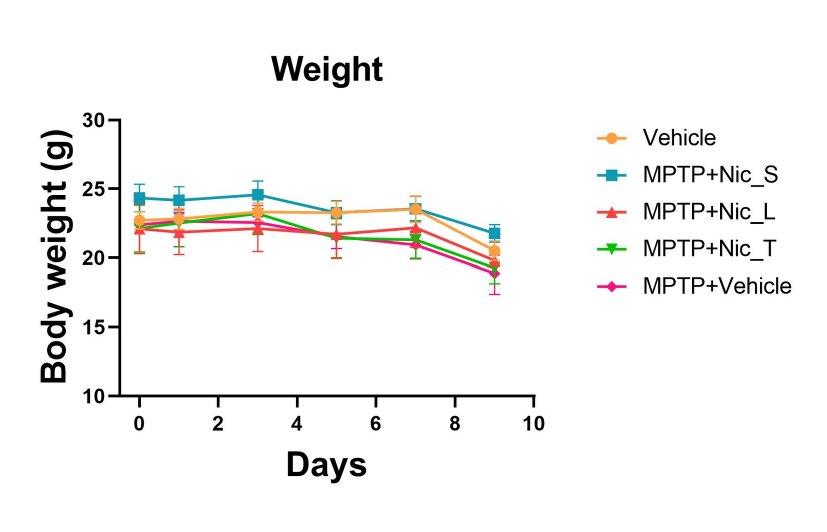

Supplement: Supplementary file 1 — Additional file 1: Figure S1. Weight curves monitored every 2 days during the in vivo experiment. n = 8 in each group. [file 12967_2024_5171_MOESM1_ESM.pptx]

## Slide 1
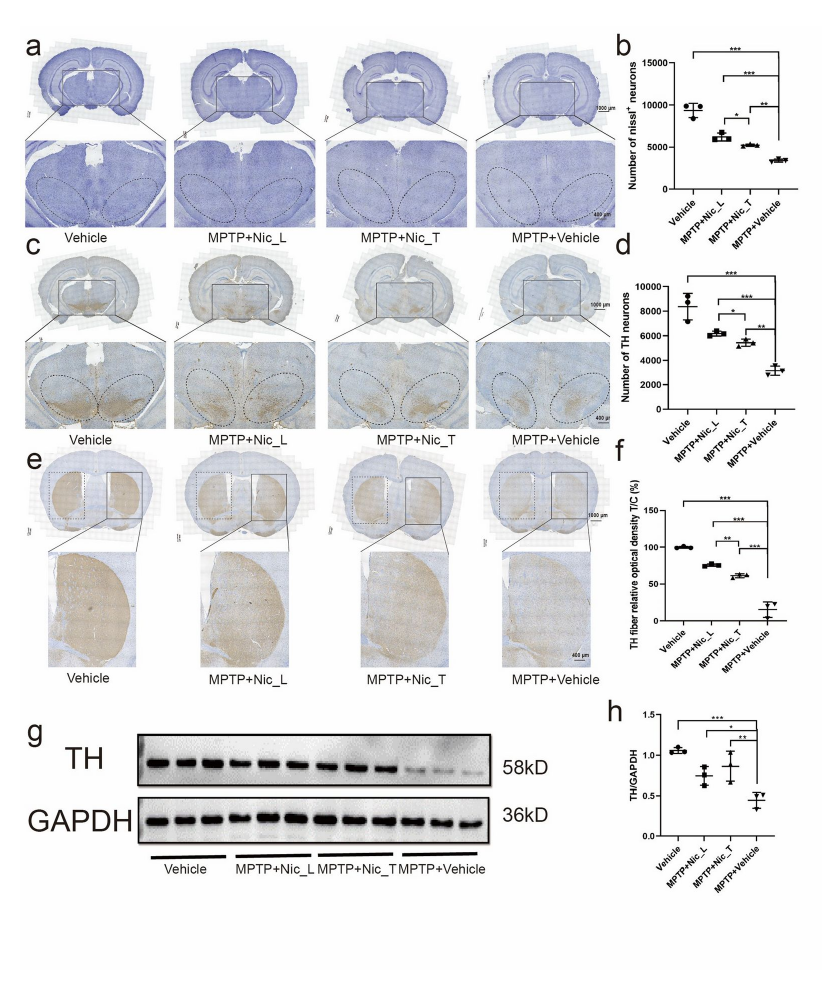

Supplement: Supplementary file 2 — Additional file 2: Figure S2. Dopaminergic neurons are preserved by nicotine treatment both in the preventative and therapeutic manner. Nissl staining (a, b) and tyrosine hydroxylase (TH) immunohistochemical (IHC) staining (c, d) of the SNpc in the coronal slice of the mouse brain. TH IHC staining of the striatum (e, f). Western blot (g, h) of TH in different treatment groups (Vehicle, MPTP + Nic_L, MPTP + Nic_T, and MPTP + Vehicle). Scale bar = 1000 μm and 400 μm. Significant differences are shown by *p < 0.05, **p < 0.01, ***p < 0.001. n = 3 in each group. [file 12967_2024_5171_MOESM2_ESM.pptx]

## Slide 1
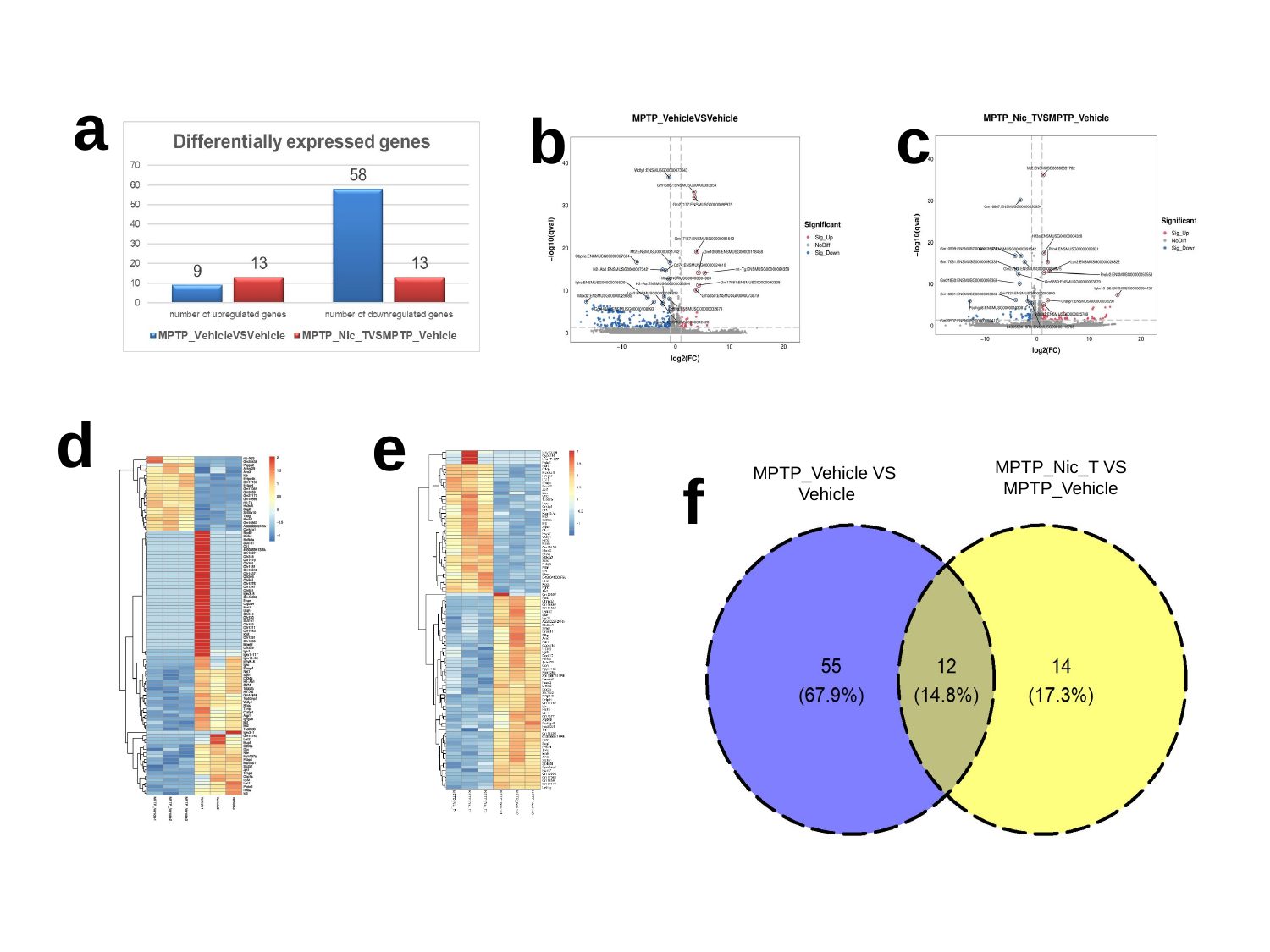

a
b
d
MPTP_Nic_T VS MPTP_Vehicle
MPTP_Vehicle VS
Vehicle
e
Prok2R
f
c

Supplement: Supplementary file 3 — Additional file 3: Figure S3. The transcriptional RNA sequence analyses. The DEGs (a) screened out by log2FC value 1.0 and FDR value < 0.05. The volcano plot (b) and heatmap plot (d) of DEGs in MPTP_Vehicle group versus Vehicle group. The volcano plot (c) and heatmap plot (e) of DEGs in MPTP_Nic group versus MPTP_Vehicle group. Venn plot (f) for DEGs in MPTP_Vehicle VS Vehicle and MPTP_Nic_T VS MPTP_Vehicle comparison group. [file 12967_2024_5171_MOESM3_ESM.pptx]

## Slide 1
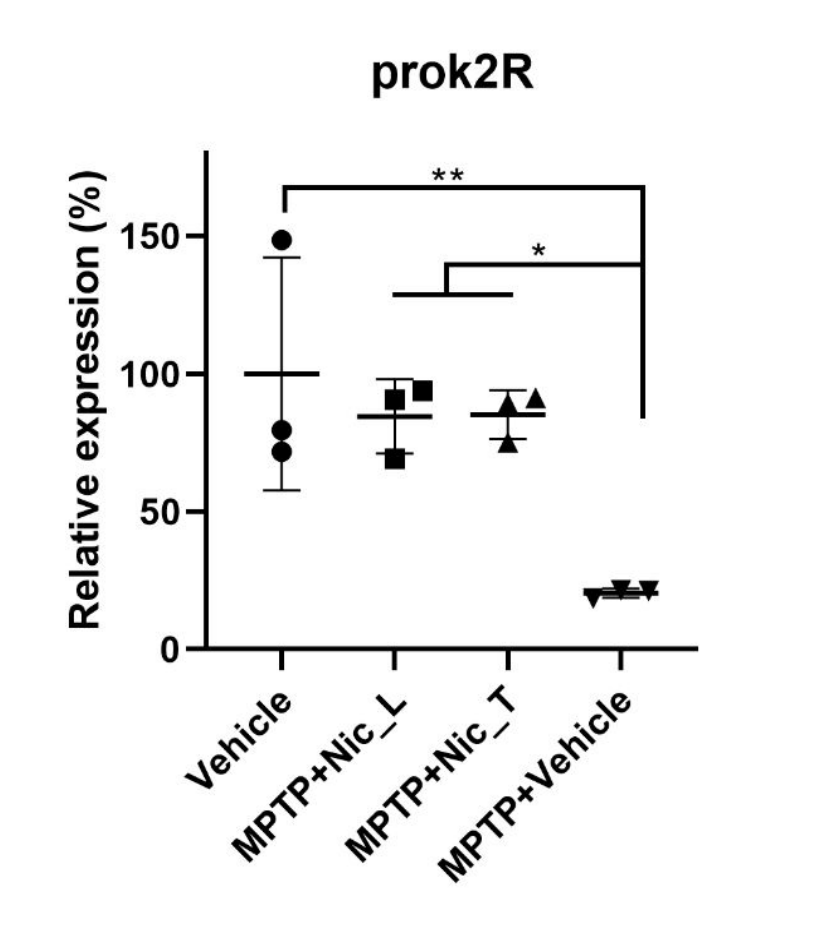

Supplement: Supplementary file 4 — Additional file 4: Figure S4. Expression levels of prok2R evaluated by reverse transcription-quantitative polymerase chain reaction. Significant differences are shown by *p < 0.05, **p < 0.01, ***p < 0.001. n = 3 in each group. [file 12967_2024_5171_MOESM4_ESM.pptx]

## Slide 1
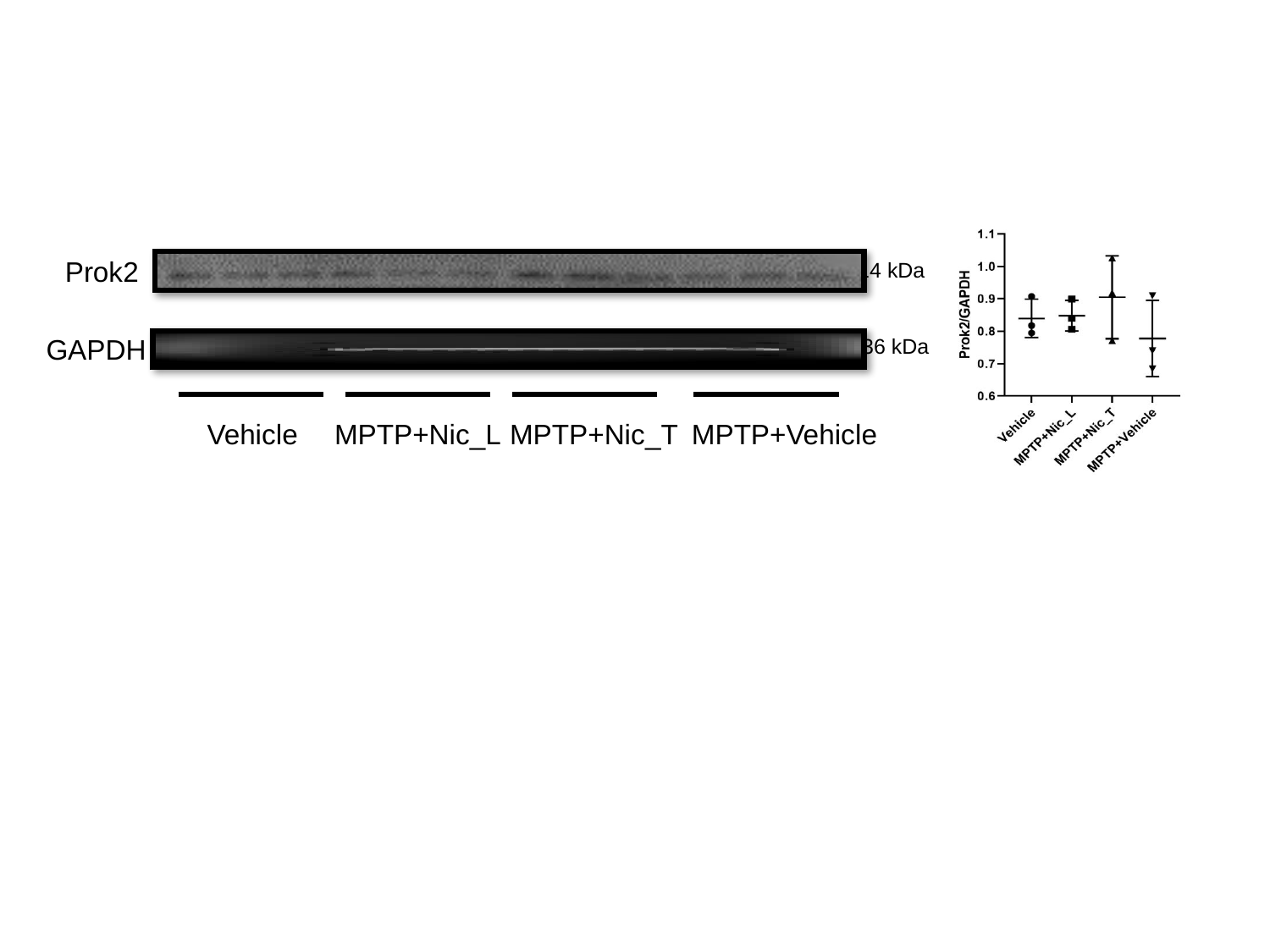

Prok2
14 kDa
GAPDH
36 kDa
Vehicle
MPTP+Nic_L
MPTP+Nic_T
MPTP+Vehicle

Supplement: Supplementary file 5 — Additional file 5: Figure S5. Expression of prok2 revealed using western blot in in vivo experiments. n = 3 in each group. [file 12967_2024_5171_MOESM5_ESM.pptx]

## Slide 1
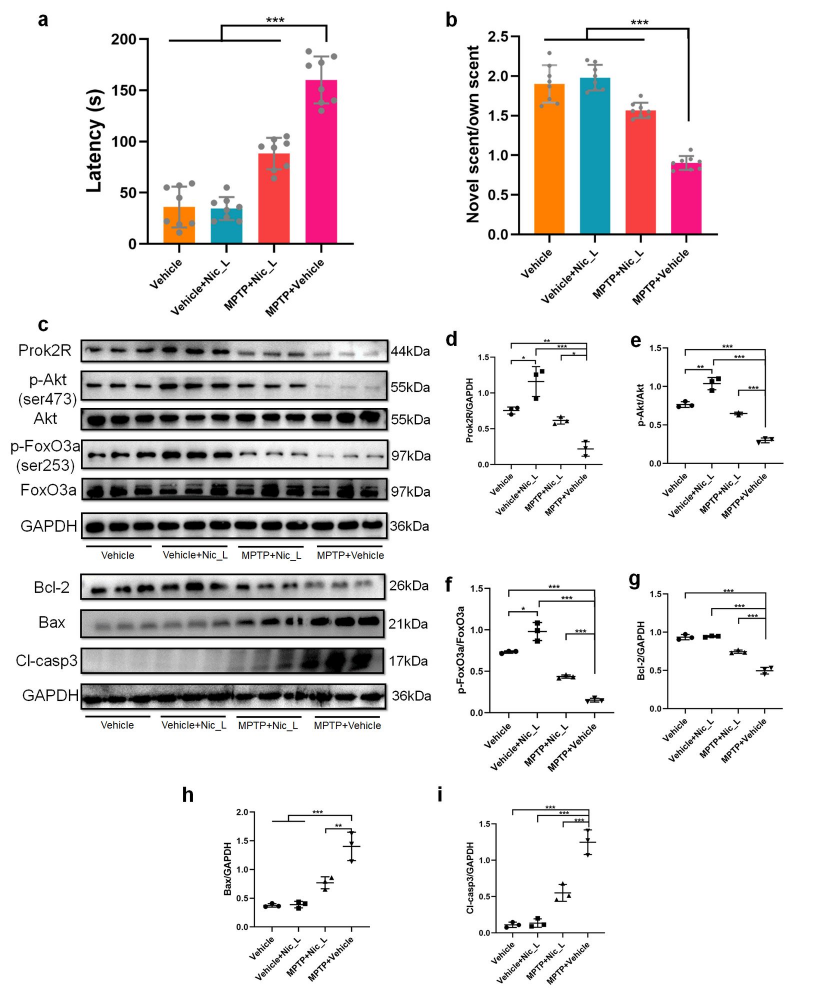

Supplement: Supplementary file 6 — Additional file 6: Figure S6. Nicotine can upregulate prok2R/Akt/FoxO3a axis thus protect olfactory from apoptotic alterations thus preserving olfactory functions in MPTP PD mice. Time latency to find the pellet (a). The ratio of time spent smelling novel scent versus own scent (b). Prok2R (c, d), p-Akt (c, e), and p-FoxO3a (c, f) expression levels evaluated by WB assay. Bcl (c, g), Bax (c, h), and Cl-caspase-3 (c, i) levels evaluated by WB assay. Significant differences are shown by *p < 0.05, **p < 0.01, ***p < 0.001. n = 3 in each group. [file 12967_2024_5171_MOESM6_ESM.pptx]

## Slide 1
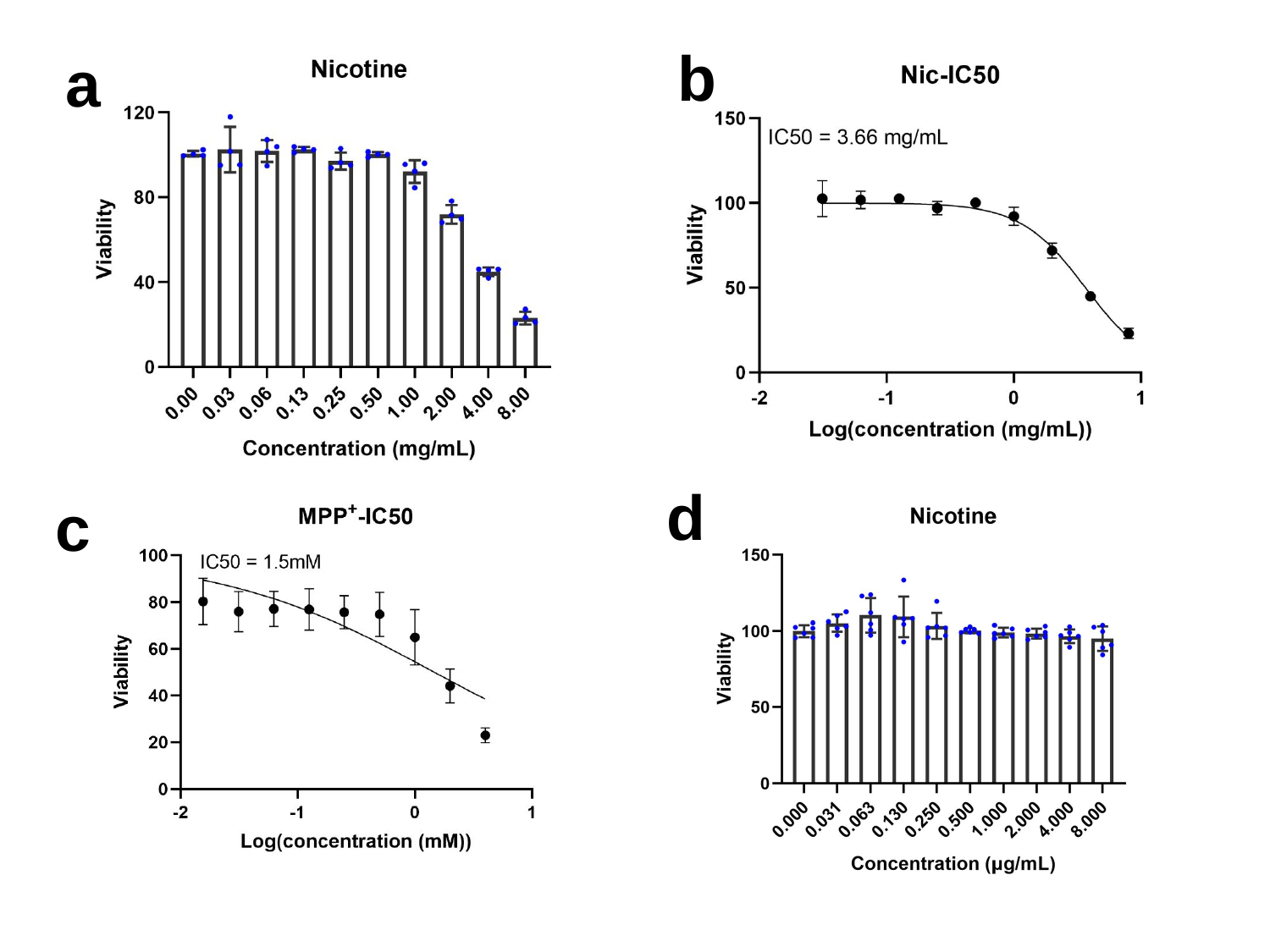

b
a
d
c

Supplement: Supplementary file 7 — Additional file 7: Figure S7. a Viability of HEK293T cells with different doses of nicotine. b IC50 calculation curve for nicotine. n = 4 in each group. c IC50 calculation curve for MPP + . d Safety evaluation experiments of nicotine for primary olfactory bulb neurons. n = 6 in each group. [file 12967_2024_5171_MOESM7_ESM.pptx]

## Slide 1
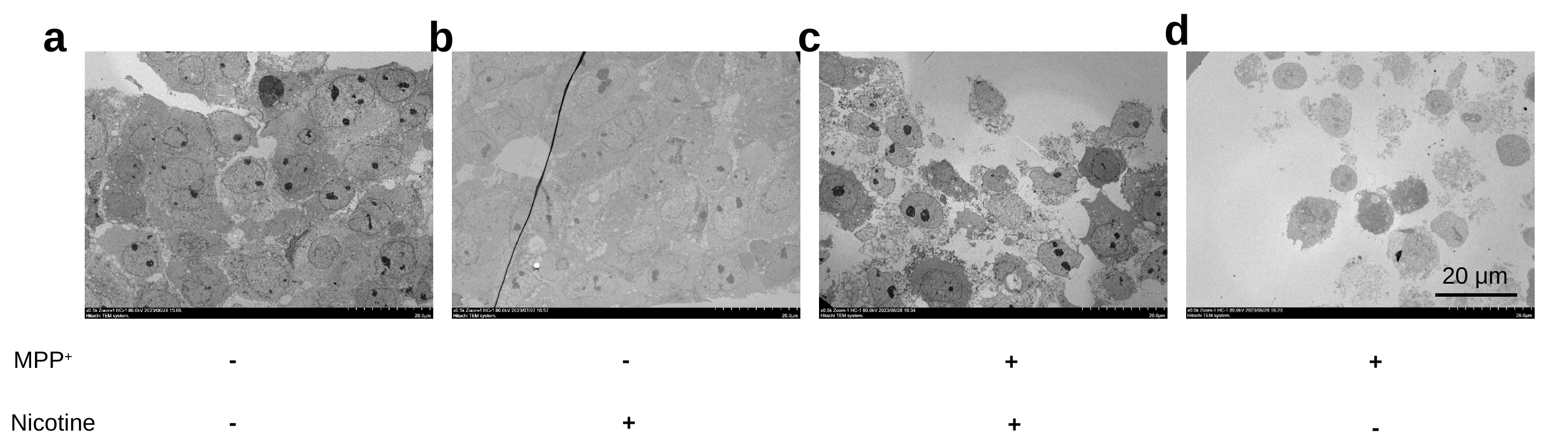

d
c
a
b
20 μm
-
-
MPP+
+
+
Nicotine
-
+
+
-

Supplement: Supplementary file 8 — Additional file 8: Figure S8. Transmission electron microscopy images of HEK293T cells from different treatment groups. Scale bar = 20 μm. [file 12967_2024_5171_MOESM8_ESM.pptx]

## Slide 1
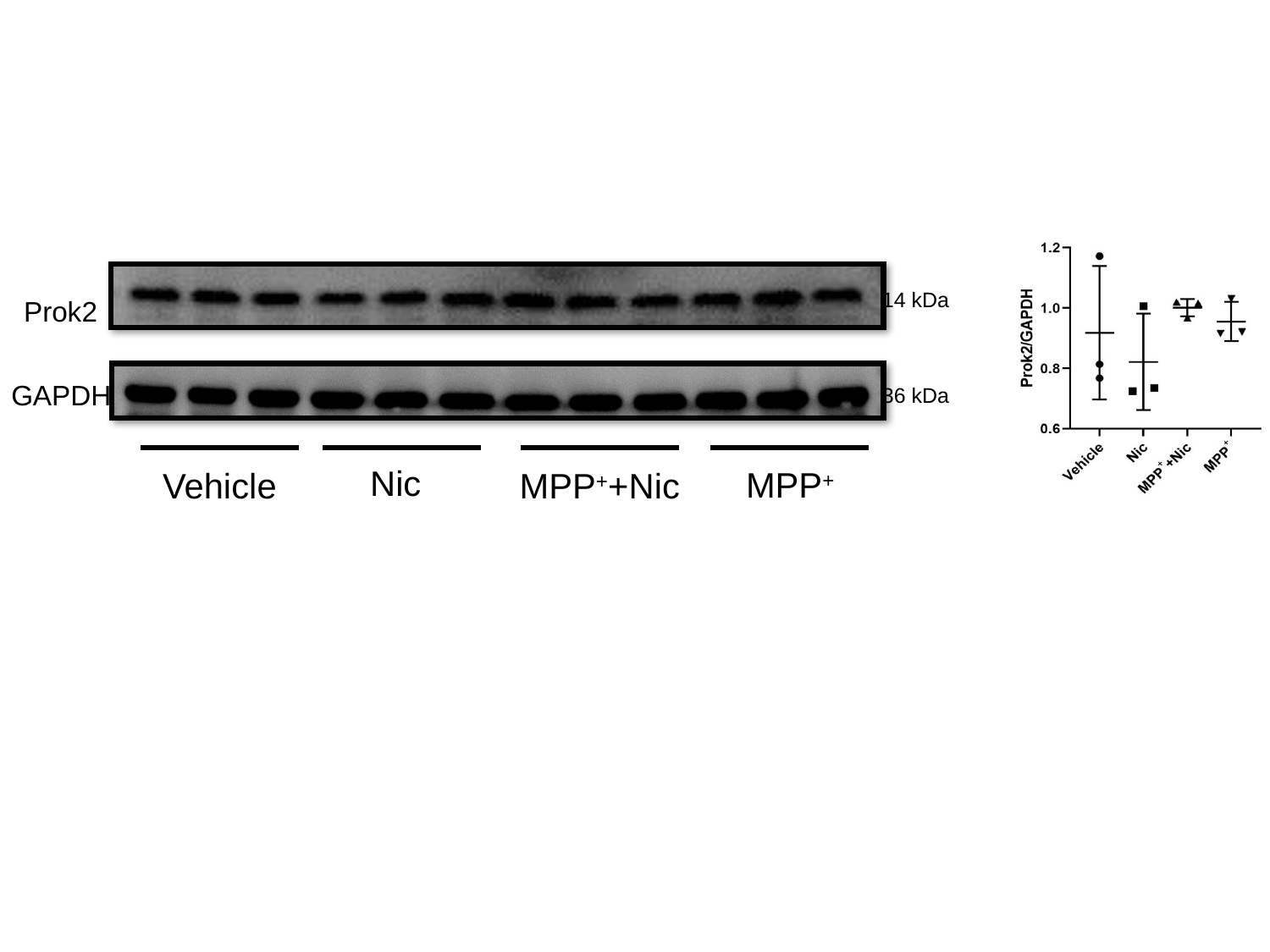

14 kDa
Prok2
GAPDH
36 kDa
Nic
MPP+
Vehicle
MPP++Nic

Supplement: Supplementary file 9 — Additional file 9: Figure S9. Prok2 expression levels in different treatment groups of HEK293T cells assessed using western blot assay. n = 3 in each group. [file 12967_2024_5171_MOESM9_ESM.pptx]

## Slide 1
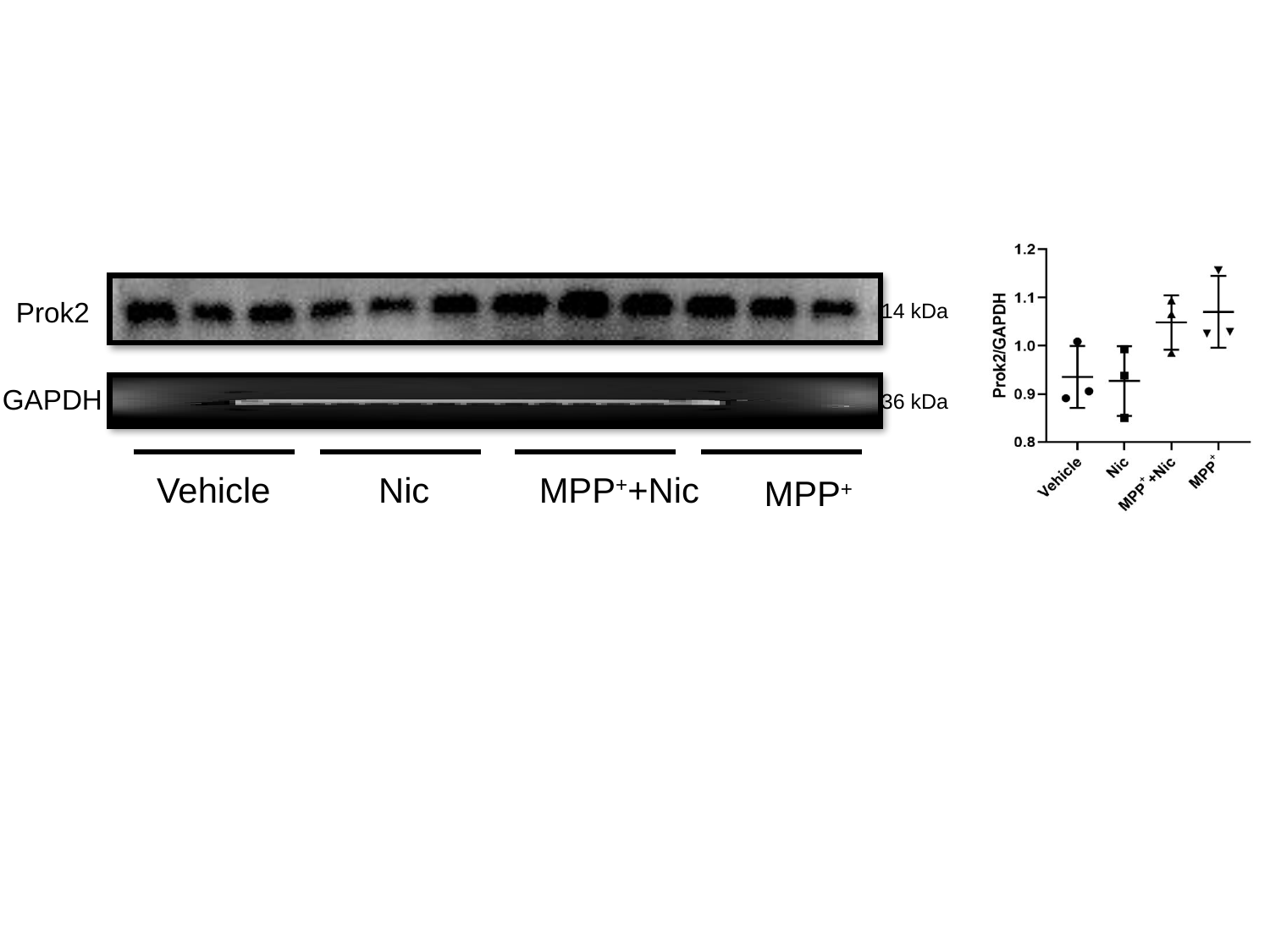

Prok2
14 kDa
GAPDH
36 kDa
Vehicle
Nic
MPP++Nic
MPP+

Supplement: Supplementary file 10 — Additional file 10: Figure S10. Prok2 expression levels in different treatment groups of primary olfactory bulb cells assessed using western blot assay. n = 3 in each group. [file 12967_2024_5171_MOESM10_ESM.pptx]
